# Conspicuously concealed: revision of the arid clade of the *Gehyra variegata* (Gekkonidae) group in Western Australia using an integrative molecular and morphological approach, with the description of five cryptic species

**DOI:** 10.7717/peerj.5334

**Published:** 2018-07-19

**Authors:** Luke Kealley, Paul Doughty, Mitzy Pepper, J. Scott Keogh, Mia Hillyer, Joel Huey

**Affiliations:** 1 Department of Terrestrial Zoology, Western Australian Museum, Welshpool, Western Australia, Australia; 2 Research School of Biology, Australian National University, Canberra, Australia; 3 School of Biological Sciences, University of Western Australia, Crawley, WA, Australia; 4 School of Natural Sciences, Edith Cowan University, Joondalup, WA, Australia

**Keywords:** Cryptic species, gecko, Integrative taxonomy, mtDNA, ND2, Pilbara, Arid zone, *Gehyra*, North West Cape, Barrow Island

## Abstract

The methods used to detect and describe morphologically cryptic species have advanced in recent years, owing to the integrative nature of molecular and morphological techniques required to elucidate them. Here we integrate recent phylogenomic work that sequenced many genes but few individuals, with new data from mtDNA and morphology from hundreds of gecko specimens of the *Gehyra variegata* group from the Australian arid zone. To better understand morphological and geographical boundaries among cryptic forms, we generated new sequences from 656 *Gehyra* individuals, largely assigned to *G. variegata* group members over a wide area in Western Australia, with especially dense sampling in the Pilbara region, and combined them with 566 *Gehyra* sequences from GenBank, resulting in a dataset of 1,222 specimens. Results indicated the existence of several cryptic species, from new species with diagnostic morphological characters, to cases when there were no useful characters to discriminate among genetically distinctive species. In addition, the cryptic species often showed counter-intuitive distributions, including broad sympatry among some forms and short range endemism in other cases. Two new species were on long branches in the phylogram and restricted to the northern Pilbara region: most records of the moderately sized *G. incognita* sp. nov. are near the coast with isolated inland records, whereas the small-bodied saxicoline *G. unguiculata* sp. nov. is only known from a small area in the extreme north of the Pilbara. Three new species were on shorter branches in the phylogram and allied to *G. montium*. The moderately sized *G. crypta* sp. nov. occurs in the western and southern Pilbara and extends south through the Murchison region; this species was distinctive genetically, but with wide overlap of characters with its sister species, *G. montium*. Accordingly, we provide a table of diagnostic nucleotides for this species as well as for all other species treated here. Two small-bodied species occur in isolated coastal regions: *G. capensis* sp. nov. is restricted to the North West Cape and *G. ocellata* sp. nov. occurs on Barrow Island and other neighbouring islands. The latter species showed evidence of introgression with the mtDNA of *G. crypta* sp. nov., possibly due to recent connectivity with the mainland owing to fluctuating sea levels. However, *G. ocellata* sp. nov. was more closely related to *G. capensis* sp. nov. in the phylogenomic data and in morphology. Our study illustrates the benefits of combining phylogenomic data with extensive screens of mtDNA to identify large numbers of individuals to the correct cryptic species. This approach was able to provide sufficient samples with which to assess morphological variation. Furthermore, determination of geographic distributions of the new cryptic species should greatly assist with identification in the field, demonstrating the utility of sampling large numbers of specimens across wide areas.

## Introduction

Cryptic species are defined as not being readily diagnosable from other species based on appearance, yet possessing a long history as independently evolving biological lineages (Bickford et al., 2007; Struck et al., 2018). In the elucidation of truly cryptic species it is perforce necessary to use molecular genetic evidence to detect them, which explains the rapid rise in cryptic species descriptions with the increase in the use of molecular genetic techniques in systematics. In hindsight, some cryptic species could have been detected using subtle differences in morphology. This is largely because taxonomists that rely on morphology tend to be conservative in their decisions and usually work on more conspicuous species first within their research programmes, thus leaving cryptic species undescribed as parts of variable species complexes.

With the rise of more affordable and rapid phylogenomic techniques to sample large numbers of loci (Lemmon, Emme & Lemmon, 2012; Peterson et al., 2012; Bragg et al., 2016) coupled with new computational analyses (Kumar & Dudley, 2007; Mirarab & Warnow, 2015), an outcome is robust phylogenies that indicate the existence of cryptic species. A problem with such studies for the systematist, however, is the trade-off between sequencing large number of genes or large numbers of individuals, as carrying out both is prohibitively expensive for most research programmes. Studies that sequence a large number of genes to document the existence of species but that have sampled a small number of individuals are problematic for defining cryptic species for two reasons.

1. Because morphological variation within a cryptic species will usually overlap that of other species in the complex (otherwise it would have likely been described previously by a morphologist), there are too few genotyped individuals with which to either tease out subtle yet consistent differences among species, or come to a sound conclusion that the species are truly cryptic with no useful diagnostic morphological characters to separate them.
2. The geographic distribution of cryptic species is poorly defined. Because morphology may be of little use to workers when collecting specimens of cryptic species in the field, understanding the geographic distribution of cryptic species can quickly narrow down the pool of potential species to compare. Although geography per se is not used in diagnoses of taxonomic works to avoid circularity, location is nevertheless a useful tool to help identify a specimen from a specific area.

**Figure 1:**
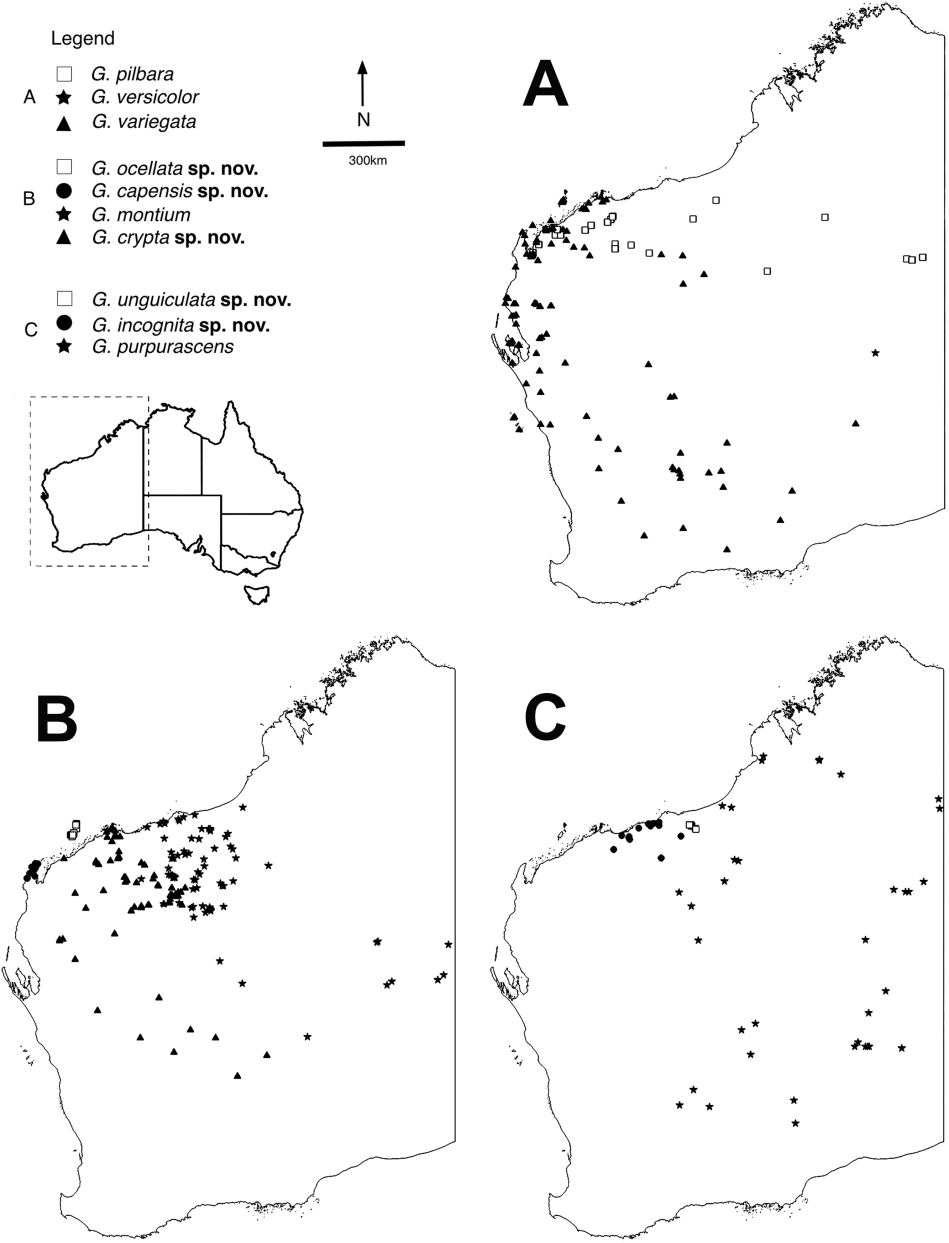
Distributions of arid clade *G. variegata* group species. Maps showing distributions of specimens identified using molecular and morphological data (Table S1). Maps were constructed in QGIS v.2.16.3 (QGIS Development Team, 2009). (A) *Gehyra variegata*, *G. pilbara* and *G. versicolor*. (B) *G. montium*, *G. capensis* sp. nov., *G. crypta* sp. nov. and *G. ocellata* sp. nov. (C) *G. purpurascens*, *G. incognita* sp. nov. and *G. unguiculata* sp. nov.

Here we present a taxonomic revision of a group of Australian geckos that qualify as a truly cryptic species complex. Since the 19th century, the small-bodied arboreal gecko species *Gehyra variegata* was believed to occur across the Australian arid zone, including cooler southern latitudes (cf. Fig. 1A). Genetic data, however, has increasingly pointed to a far more complex picture. Chromosome evidence from the 1970s and 1980s (King, 1979; Moritz, 1986) indicated several cryptic forms, some of which were described as new species. Beginning with the study of Sistrom et al. (2009), molecular data have unearthed a wealth of phylogenetic diversity in the arid group, while studies in parallel on northern species have also contributed to *Gehyra* diversity in Australia (Doughty et al., 2012, 2018; Oliver et al., 2016; Bourke et al., 2017; Moritz et al., 2018). Further studies of Sistrom and colleagues (Sistrom et al., 2012; Sistrom, Donnellan & Hutchinson, 2013; Hutchinson et al., 2014) began to clarify the distribution of true *G. variegata* (Duméril & Bibron, 1836) (type location = Shark Bay in Western Australia (WA)), along with many other cryptic forms, several occurring in the western arid zone and coastal regions. As a result of these studies, *G. lazelli* (Wells & Wellington, 1985) was redescribed for Flinders Ranges and Eyre Basin populations in South Australia and a new species, *G. versicolor* Hutchinson et al., 2014, was applied to the eastern arid zone, further reducing the extent of *G. variegata*’s distribution. In addition, *G. montium* Storr, 1982 was found to occur in the Pilbara, greatly extending its range across the western arid zone from the type location of the Central Ranges (cf. Fig. 1B). Other lineages were left undescribed pending further work (M. Sistrom, M. Hutchinson, 2014, personal communication). More recently, Ashman et al. (2018) carried out a detailed phylogenomic study of all Australian *Gehyra* using exon capture techniques. They found strong genetic evidence for the previously described species and also for multiple species within the residual *G. variegata*, including many forms from the western arid zone, especially the Pilbara region.

There are several other problems when working with *Gehyra* species (P. Doughty, 2018, personal observation). They are abundant in natural settings and specimens are frequently collected, creating a ‘specimen burden’ to systematists who wish to come to terms with the morphological variation within and among lineages. In Australian collections, there are over 25,000 specimens of *Gehyra*, including over 10,000 in the Western Australian Museum alone. However, because they are commonly encountered and also very fast and agile lizards, they are not photographed as often as more striking lizards such as dragons and knob-tailed geckos that are more easily posed. Individuals are also capable of changing shade from pale to dark depending on the time of collection and if they are kept in calico bags prior to photographing the next day (P. Doughty, 2018, personal observation). Furthermore, patterns and colours of voucher specimens fade rapidly after being immersed in ethanol, greatly deteriorating these potentially important diagnostic characters (Sistrom, Donnellan & Hutchinson, 2013; P. Doughty, 2018, personal observation).

In this study, we rely on the works of Sistrom, Donnellan & Hutchinson (2013) and Ashman et al. (2018) that used multiple mtDNA and especially nDNA loci, as providing the necessary evidence for the existence of several new species in the arid clade within the *G. variegata* group that we treat here (see Table 1 for terminology for groups within Australian *Gehyra*). We consider all species in this group, except *G. punctata*.

**Table 1:** Composition of taxonomic groups within Australian *Gehyra*.

1. *australis* group (*australis*, *borroloola*, *catenata*, *dubia*, *koira*, *pamela*, *robusta*)
2. *Relict species (*xenopus*, *spheniscus*, *lazelli*, *moritzi*, *pulingka*)
3. *variegata* group:
a. *nana* clade (*nana*, *girloorloo*, *granulum*, *kimberleyi*, *multiporosa*, *occidentalis*, *paranana*, *pluraporosa*, *pseudopunctata*)
b. **Arid clade
i. *variegata* species-group (*variegata*, *pilbara*, *minuta*, *montium*, *versicolor*, *capensis* sp. nov., *crypta* sp. nov., *ocellata* sp. nov.)
ii. *purpurascens* species-group (*purpurascens*, *einasleighensis*, *incognita* sp. nov.)
iii. *unguiculata* sp. nov.
iv. *punctata*

**Notes:**

Names used for various groups of Australian *Gehyra* species discussed in this and other recent papers (Ashman et al., 2018; Doughty et al., 2018).

* Species that do not fall neatly into either the *australis* or *variegata* groups, and tend to have relictual distributions.

To remedy the two problems outlined above (i.e. few genotyped individuals to compare morphology and establish geographic ranges) we present information derived from sequencing 656 individuals from WA, which we combined with an additional 566 *Gehyra* sequences available on GenBank. Our data provided sufficient numbers of specimens with which to evaluate morphology for comparisons and descriptions, as well as providing basic distributions for all species over a large area of the Australian arid zone. Our study outlines a means to integrate phylogenomic work, widespread mtDNA screens and morphology to resolve the systematics of a difficult cryptic species group. We conclude with a taxonomic section describing five new species, and include much of the comparative information for previously described species in the Supplemental Materials.

## Materials and Methods

### Molecular genetics

Ashman et al. (2018) took a phylogenomic approach to *Gehyra* diversity, sampling 547 loci across 42 candidate species to produce a resolved tree of relationships. However, only 1–4 individuals per lineage were assessed with several methods, including RAxML, BPP, ASTRAL and *Beast2. Based on previous analyses of *G. variegata*-like specimens, we believed that there would be high similarity among many of the arid clade species, and accordingly sampled widely within *G. variegata*, *G. montium*, *G. pilbara* Mitchell, 1965 and *G. purpurascens* Storr, 1982. We tended to not presume species identifications were correct, as the aim was to resolve a cryptic species complex, therefore making prior identifications suspect. We also drew from misidentified specimens that were the focus of other projects (Hutchinson et al., 2014; Oliver et al., 2016).

The goal of widespread DNA sequencing was to determine the distribution of species, and to identify putative specimens upon which morphological analysis could be carried out. We sequenced a ∼1,100 base pair region of the mitochondrial gene *ND2* for 656 samples, focusing on *G. variegata*-like specimens. To this we added published GenBank sequences for an additional 566 individuals. Genomic DNA was extracted and the *ND2* region was amplified following Pepper, Doughty & Keogh (2006), with sequencing carried out at the Australian Genome Research Facility. For newly prepared specimens, sequences and workflows were managed in the Geneious software package (version 7.1.5) (Kearse et al., 2012), using the LIMS Biocode plug-in (http://www.mooreabiocode.org). Sequences were checked for contamination and errors using BLAST and translation into amino acids. The final dataset (*N* = 1,222) was aligned using the MAAFT plugin (Katoh et al., 2002) in Geneious, with the default settings. A phylogenetic tree was built using the RaxML tool (Stamatakis, 2006) in the Cipres environment (Miller et al., 2015), using the default settings with 100 bootstrap replicates and the GTR+G substitution model. The tree was rooted on the gekkonid species *Heteronotia binoei* (Gray, 1845).

From this more exhaustive dataset, and through comparison with previously published datasets, specimen examinations, geography and Ashman et al. (2018), lineages corresponding to species were identified. Identifications were then updated, and species distributions could be visualised. From this, a smaller dataset was selected for morphological analysis, and for calculating diagnostic nucleotides. For the latter, we carefully selected sequences that encompassed the phylogenetic diversity of each species, with short sequences and sequences from specimens inadequate for morphology ignored. Up to 31 sequences were selected for each species.

**Figure 2:**
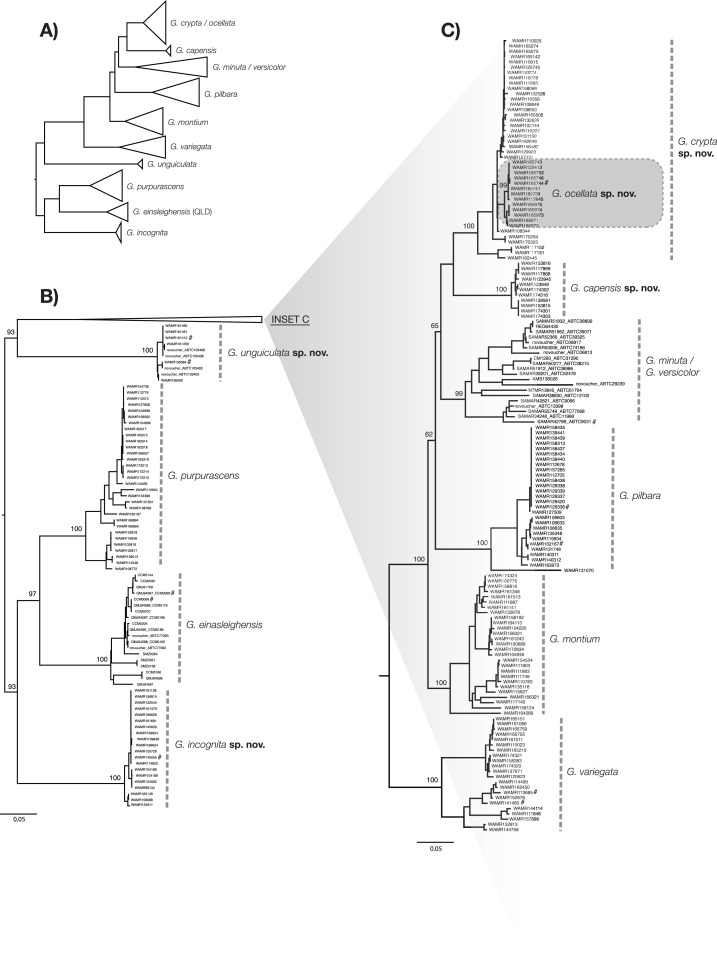
ML phylogenetic tree of reduced specimen dataset, based on *ND2* gene, for *Gehyra* species considered here. (A) Complete tree for the arid clade of *Gehyra variegata* group members considered here, with species collapsed. (B) Expanded tree for *G. unguiculata* sp. nov., *G. purpurascens*, *G. einasleighensis* and *G. incognita* sp. nov. (C) Expanded tree for *G. crypta* sp. nov., *G. ocellata* sp. nov., *G. capensis* sp. nov., the *G. minuta–versicolor* complex, *G. pilbara*, *G. montium* and *G. variegata*. For (B) and (C), specimens are described by voucher registration numbers where available, any other registration number (e.g. field codes and tissue codes), and the GenBank registration number. Bootstrap values are provided for species and relationships among species. # = Also sequenced in Ashman et al. (2018).

To calculate diagnostic nucleotides for each species, we followed the rationale outlined in Jörger & Schrödl (2013) and the method implemented in Framenau et al. (in press). Using the R package ‘Spider’ (Brown et al., 2012), we identified nucleotide changes that are fixed within, and unique to each species (so called ‘single pure characters’). The dataset that formed the basis of this analysis was comprised of 230 sequences from five new species and seven previously described species: *G. variegata*, *G. pilbara*, *G. montium*, *G. purpurascens*, *G. minuta* King, 1982, *G. versicolor* and *G. einasleighensis* Bourke et al., 2017. Owing to an inability to easily identify the phylogenetic boundary between lineages of *G. minuta* and *G. versicolor* (Sistrom, Donnellan & Hutchinson, 2013), both species were included as a single group in the analysis. Also, due to potential historical introgression between two of the new species (see discussion), they were analysed both as a single group as well as separately to create ‘nested’ diagnostic nucleotides. This dataset was also used to generate a phylogenetic tree to visualise the data following the same method detailed above (Fig. 2). The tree was rooted using *G. purpurascens*, *G. einasleighensis* and a new species to recover a similar topology to Ashman et al. (2018). As the aim of this study was not to recover the true evolutionary history of the group (Sistrom, Donnellan & Hutchinson, 2013; Ashman et al., 2018), we felt this visualisation best summarised the data and linked lineages reported here to those in previous studies.

### Morphometrics

We measured 258 specimens in total (Table S1). Almost all specimens had been sequenced for *ND2* providing an independent estimate of species identity. Given the possibility of hybrids between closely related lineages, if there was a strong mismatch between the morphology of a specimen and the majority of other specimens, then the outlying specimen was not included in the morphological analysis. Juveniles were excluded, with gravid females and males with fully developed pre-cloacal pores providing an indication of size at maturity. Genotyped but poorly preserved specimens were not chosen for morphological analyses, so long as there were at least 30 individuals included for each lineage (although some lineages had less than 30 genotyped individuals to measure).

**Figure 3:**
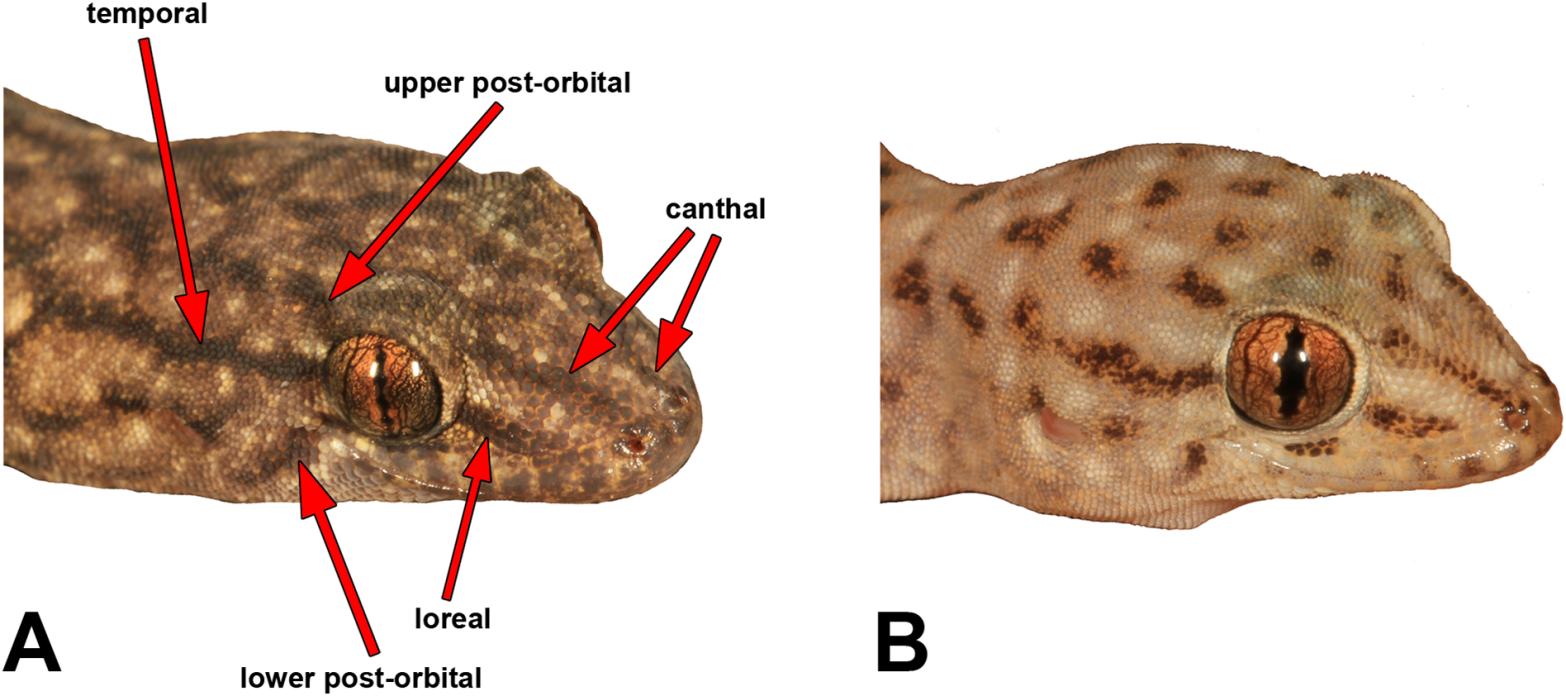
Head stripe scoring guide. (A) Head of *Gehyra montium* (WAM R175382), showing the stripes scored with terminology in the analysis of patterning. (B) Head of *G. capensis* sp. nov. (WAM R174314) showing presence of canthal, loreal and temporal stripes (albeit weaker than in (A)), with post-orbital stripes absent (but with elements forming spots on the head). Photo credit—R.J. Ellis.

We measured most of the characters included in previous *Gehyra* descriptions, namely Sistrom et al. (2009), Doughty et al. (2012) and Hutchinson et al. (2014). Table S2 provides definitions and abbreviations of the characters measured. We measured SVL and TailL to the nearest 0.5 mm with a rule, and other measurements were carried out with Mitutoyo electronic callipers to the nearest 0.1 mm. We made note of the presence and strength of five different head stripes, which are illustrated in Fig. 3.

In Table S2, we present a large summary table with all characters measured, separated by lineage and by males and females. From this very large table, we extracted meaningful differences among taxa and present these in a summary table (Table 2), along with notes on pattern elements and colour. The taxonomy section below features the five new species described, but with redescriptions of other *G. variegata* group arid clade members in the Supplemental Material. In the taxonomy section below, all locations are from WA unless noted otherwise. Collection of specimens in WA was undertaken through a licence issued to the WA Museum by the Department of Parks & Wildlife (WA) under the Animal Welfare Act 2002 (‘Licence to use animals for scientific purposes,’ No. U 10/2015–2018), and a collecting permit to take native fauna (08-000214-2).

**Table 2:** Summary of diagnostic traits.

Species	SVL mean (range)	4TLam	Internarials	Relative post-nasal size	SnoutL/SVL	Pairs of chin shields	Infralabial notched	Pre-cloacal pores	Dorsal, head stripe and ventral patterns
*G. capensis* sp. nov.	40.2 (34.0–46.0)	6.3 6–18 (72%) 7–7 (28%)	0.72 0–7 (28%) 1–18 (72%)	Lower > upper	High (0.12)	2	2nd	10.8 (9–12)	**Pinkish-grey**; dark brown irregular spots or short bars with numerous smaller white spots. Canthal, loreal and temporal stripes present; **no post-orbital stripes**. Ventrum without stippling.
*G. crypta* sp. nov.	47.7 (41.0–56.5)	6.6 6–14 (47%) 7–15 (50%) 81 (3%)	0.80 0–6 (20%) 1–24 (80%)	Equal	Medium (0.11)	2	2nd or 3rd	12.4 (10–16)	In preservative, light grey to dark brown; **highly variable pattern**, from isolated dark and pale bars to dark network with white spots to patternless. Head stripes present, but with **lower post-orbital stripe at most a spot**. Ventrum with medium to dense stippling.
*G. ocellata* sp. nov.	40.5 (32.0–49.0)	6.2 6–15 (83%) 7–3 (17%)	0.39 0–11 (61%) 1–7 (39%)	Lower > upper	Medium (0.11)	2	2nd	11.1 (10–12)	**Light to medium reddish-brown**; numerous pale spots with brown irregular markings variable expressed. **Head stripes poorly defined to absent.** Ventrum with little to no stippling.
*G. incognita* sp. nov.	45.2 (36.0–52.0)	6.0 5–2 (8%) 6–21 (88%) 7–1 (4%)	0.54 0–11 (46%) 1–13 (54%)	Lower ≥ upper	Medium (0.11)	2	2nd	12.0 (10–16)	In preservative, medium grey to dark brown; poorly contrasting back pattern of **small dark and pale spots**, occasionally forming bars or networks. Head stripes present. Ventrum with heavily stippled.
*G. unguiculata* sp. nov.	37.3 (34.5–39.0)	6.1 6–8 (88%) 7–1 (12%)	0.67 0–3 (33%) 1–6 (67%)	Lower ≥ upper	Medium (0.11)	2	2nd	12.3 (11–13)	In preservative, light tan to medium brown; dorsal markings **dark crescent-shaped bars with pale spots posteriorly**; crown with pale white spots. **Head stripes usually poorly defined.** Lateral edges of ventrum with some stippling, central area devoid of stippling.
*G. variegata*	43.5 (37.0–52.5)	6.3 6–24 (77%) 7–5 (16%) 8–2 (7%)	0.48 0–16 (52%) 1–15 (48%)	Equal	Medium (0.11)	2 or 3	2nd	11.5 (10–15)	Light to dark grey-brown; **dark network** of variably connected bars and lines with pale posterior edges or spots. Well-defined head stripes. Ventrum heavily stippled.
*G. versicolor*	49.5 (40.5–58.0)	7 7–8 (100%)	0.63 0–3 (37%) 1–5 (63%)	Lower > upper	Medium (0.11)	2	2nd	14 (13–15)	As for *G. variegata*, except in general pattern with lesser contrast (Hutchinson et al., 2014).
*G. minuta*	34.4 (24.0–40.0)	6.5 6–12 (52%) 7–11 (48%)	0.70 0–7 (30%) 1–16 (70%)	Lower >> upper	High (0.12)	2	2nd	12 (10–14)	**Pinkish to reddish-brown**; scattered short dark bars and pale spots scattered over dorsum. Canthal, loreal and temporal stripes present; **no post-orbital stripes**. Ventrum with moderate stippling.
*G. pilbara*	43.2 (33.5–51.5)	6.9 6–3 (10%) 7–26 (87%) 8–1 (3%)	0.43 0–17 (57%) 1–13 (43%)	Lower >>upper	Low (0.10)	2	2nd	12.5 (10–14)	Dull **orange to reddish-brown**, dorsum with scattered dark brown spots or bars with more numerous white spots not in contact. **Weak canthal and temporal stripes.** Ventrum with little to no stippling.
*G. montium*	44.7 (35.5–49.5)	6.7 6–8 (27%) 7–22 (73%)	0.90 0–3 (10%) 1–27 (90%)	Lower ≥ upper	Medium (0.11)	2 or 3	2nd or 3rd	12 (10–15)	Greyish-brown; **short brown bars** in contact with pale white markings; highly variable. Well-defined head stripes. Ventrum heavily stippled.
*G. purpurascens*	53.6 (45.5–67.0)	7.4 6–1 (3%) 7–15 (50%) 8–14 (47%)	0.83 0–5 (17%) 1–25 (83%)	Lower ≥ upper	Medium (0.11)	2 or 3	2nd or 3rd	9.7 (8–11)	Purplish-grey to brown; **dark network** of thin lines with white spots. Canthal and loreal stripes present, **temporal and post-orbital stripes variably expressed**. Ventrum with little to moderate stippling.

**Note:**

Summary of quantitative and qualitative morphological traits and pattern and colouration that vary among members of the arid clade of the *variegata* group treated here. Bold indicates potentially useful diagnostic characters that differ from one or more species. Abbreviations and explanations of characters measured are presented in Table S2.

The electronic version of this article in portable document format will represent a published work according to the International Commission on Zoological Nomenclature (ICZN), and hence the new names contained in the electronic version are effectively published under that Code from the electronic edition alone. This published work and the nomenclatural acts it contains have been registered in ZooBank, the online registration system for the ICZN. The ZooBank LSIDs (Life Science Identifiers) can be resolved and the associated information viewed through any standard web browser by appending the LSID to the prefix http://zoobank.org/. The LSID for this publication is: urn:lsid:zoobank.org:pub:D3B2FF0E-C0D7-4E52-B3D5-CC37A60DC8B6. The online version of this work is archived and available from the following digital repositories: PeerJ, PubMed Central and CLOCKSS.

### Species concept

Here we employ a lineage-based species concept (Frost & Hillis, 1990; De Queiroz, 1998, 2007) and use the different lines of evidence to test whether groups represent independently evolving historical lineages. To do this we sought multiple corroborating lines of evidence for us to recognise species, with the understanding that recent genetic introgression may lead to patterns in the mtDNA not recovered from the slower-evolving nDNA data of Ashman et al. (2018). When there was conflict between the genetic data sets, morphology provided an independent line of evidence to test for species status. When there was little evidence from morphology, particularly owing to wide overlap among characters, we looked for strong genetic patterns in both the mtDNA and nDNA data sets. Ultimately, all species recognised here differed in at least two independent data sets, and most in all three. In addition, geography was also congruent with our taxonomic decisions, resulting in sound taxonomic decisions made based on the main signal of evidence from all four data sets.

## Results

For ease of explanation owing to the large number of taxa discussed here, we use the ultimate species names in the descriptions below rather than code names based on location or genetic lineage.

### Molecular genetics

The previous genetic studies of Sistrom et al. (2009), Sistrom, Donnellan & Hutchinson (2013), Pepper, Doughty & Keogh (2013) and Ashman et al. (2018) provided strong evidence for the existence of multiple cryptic species within the arid clade of the *G. variegata* group. Our focus here on sequencing the mtDNA gene *ND2* was to assign numerous specimens to lineages to enable a morphological and geographic appraisal of the described and undescribed forms. Accordingly, we review our results in light of the combined genetic data and morphological appraisal.

The exhaustive genetic dataset (*N* = 1,222; Table S1) successfully linked hundreds of incorrectly identified and unidentified specimens to lineages identified in previously published studies, and to existing and new species. The reduced phylogeny (Fig. 2) shows the five new species form lineages on long branches, distinct from other species in the arid clade of the *G. variegata* group. This was not the case for *G. crypta* sp. nov., however, which was rendered paraphyletic by *G. ocellata* sp. nov. This conflicts with Ashman et al. (2018), who recovered these lineages as distinct, and with *G. ocellata* sp. nov. (their ‘variegataB2’) more closely related to *G. capensis* sp. nov. (‘variegataB1’) than to *G. crypta* sp. nov. (‘variegataB3’). Using these data, and updated identifications based on new morphological characters (see below), the distributions of these species can be visualised and are shown in Fig. 1.

Molecular diagnostic nucleotides (Table 3; raw data and GenBank accession numbers provided in Supplemental Material) were recovered for each species compared to all other species, except for *G. crypta* sp. nov. owing to the aforementioned paraphyly of the species relative to *G. ocellata* sp. nov. in the mtDNA data. As such, we calculated a nested set of characters, comparing *G. crypta* sp. nov. and *G. ocellata* sp. nov. to all other species, and then these species against each other. Notably, *G. ocellata* sp. nov. had a single diagnostic nucleotide which diagnosed it relative to all other species in the analysis.

**Table 3:** Diagnostic nucleotides for species of the arid clade of the *Gehyra variegata* group.

**Species pairs**
*G. crypta* sp. nov.–*ocellata* sp. nov. (*N* = 42), compared to all other species336(C), 366(C)
*G. minuta*–*versicolor* (*N* = 20), compared to all other species420(T/C)
**Single species**
*G. capensis* sp. nov. (*N* = 11), compared to all other species234(C), 489(G), 643(C), 757(G), 841(G)
*G. ocellata* sp. nov. (*N* = 13), compared to all other species702(G)
*G. crypta* sp. nov. (*N* = 29), compared to all other speciesN/A
*G. incognita* sp. nov. (*N* = 20), compared to all other species232(T), 243(C), 274(G), 275(A), 319(A), 390(C), 399(T), 454(C), 499(A), 511(C), 540(C), 594(T), 598(C), 628(T), 630(G), 669(G), 670(C)
*G. unguiculata* sp. nov. (*N* = 10), compared to all other species156(C), 222(G), 262(G), 299(T), 427(A), 477(C), 490(A), 491(C), 630(C), 631(T), 639(C), 654(A), 661(C), 696(A), 735(G), 762(T), 783(C), 786(C), 810(T), 892(G)
*G. variegata* (*N* = 22), compared to all other species235(G), 330(C), 432(A), 458(C), 582(A), 705(C)
*G. pilbara* (*N* = 28), compared to all other species219(T), 734(C), 781(A)
*G. montium* (*N* = 27), compared to all other species309(A), 828(C)
*G. purpurascens* (*N* = 31), compared to all other species34(G), 263(C), 445(T), 845(C)
*G. einasleighensis* (*N* = 19), compared to all other species595(G), 744(A), 912(C), 921(A), 967(G)
**Pairwise comparisons**
*G. crypta* sp. nov. (*N* = 29) compared to *G. ocellata* sp. nov. (*N* = 13)306(C), 702(A), 1,003(A), 1,004(G)
*G. ocellata* sp. nov. (*N* = 13) compared to *G. crypta* sp. nov. (*N* = 29)306(T), 702(G), 1,003(G), 1,004(C)

**Note:**

Nucleotides for each focal species compared to all other species, species pairs compared to all other species and for select pairwise comparisons. All numbers are relative to the first base of GenBank accessioned sequence JX946961.

### Morphological analyses

A rigorous series of measurements, observations and genetic results provided some illumination of the number of species in the group and the phenotypic variation within and among lineages.

Table S2 presents detailed meristic character summaries of all species included here, separated by sex. Many of the characters measured did not differ among species owing to morphological conservatism or wide overlap among characters. However, Table 2 presents the main characters that did appear to vary reliably among species, including pattern. Meristic characters that exhibited potentially useful diagnostic variation were body size, relative snout length, presence and number of internarial scales, number of fourth toe lamellae and pre-cloacal pores in males. Other potential variable traits, albeit with more overlap among species, were the number of labial scales and chin shields, and whether the second or third infralabial was notched by the par infralabial row. For pattern, potentially useful elements for diagnostic traits were greyish vs reddish background colour, patterns of dark and pale lines vs spots on the dorsum, presence and strength of head stripes (Fig. 3) and degree of stippling on the ventrum.

Within the arid clade of the *G. variegata* group (Table 1), several taxa are morphologically diagnosable whereas other taxa show wide overlap of characters among species. The species *G. pilbara* and *G. ocellata* sp. nov. are the most distinctive superficially owing to their orange or reddish colouration. *G. pilbara* is also distinctive in possessing an extremely shortened snout relative to other *Gehyra*, a likely adaptation to living in termite mounds and concomitant myrmecophagy. Similar to these species is *G. capensis* sp. nov. that also has a dorsal pattern consisting of isolated dark and pale spots or bars and is relatively small-bodied, but lacks the reddish hues of the other species. Although *G. capensis* sp. nov. resembles *G. minuta*, it is restricted to the North West Cape whereas the latter species only occurs in central Northern Territory.

The two species *G. variegata* and *G. versicolor* are nearly phenotypically identical, but possess different chromosome morphology (Hutchinson et al., 2014). The wide-ranging *G. montium* occurs from the Central Ranges across the western deserts and into the eastern Pilbara (Fig. 1B), with significant overlap of morphology with other arid clade members. Another difficult species is *G. crypta* sp. nov., which shows wide overlap of morphology in characters and pattern and colouration with *G. montium* (and hence other species as well), and also co-occurs geographically with several other species, making it one of the most difficult species to distinguish.

The largest species within the *G. purpurascens* species-group (Table 1) is *G. purpurascens* itself, which also possesses relatively few pre-cloacal pores in males, a dark network on a purplish dorsum and is arboreal. In contrast, *G. einasleighensis* is saxicoline, has a dorsal pattern with isolated spots that is more typical of rock-dwelling species and occurs in northern Queensland. The cryptic species *G. incognita* sp. nov. has a fairly non-descript appearance in preservative (no images in life are yet available) and overlaps phenotypically with several other forms and occurs in the north-western Pilbara (Fig. 1C), rendering confident identification difficult without genetic analyses.

The short range endemic, *G. unguiculata* sp. nov., is a very small-bodied species with a unique dorsal pattern that is restricted to a small area in the northern Pilbara, and is adjacent to the distributions of *G. purpurascens*, *G. incognita* sp. nov. and is sympatric with *G. montium*. In the study of Ashman et al. (2018), this species was found to be included within the *G. purpurascens* species-group in some analyses and outside of this group in others; we maintain it as a unique lineage within the arid clade here (Table 1).

## Discussion

Following the previously published datasets, we recovered evidence for multiple genetic lineages in the arid clade of the *G. variegata* group. The ‘broad coverage—single gene sequencing’ approach enabled us to assign hundreds of specimens to species, allowing for an accurate appraisal of species distributions, and making the morphological description of these species possible (see below). Furthermore, diagnostic mutations could be calculated for each species, ensuring representative genetic diversity was included in the analysis, and allowing the diagnosis of cryptic species pairs (e.g. *G. crypta* sp. nov. and *G. montium*).

However, one species pair was not recovered using the single gene approach. *G. crypta* sp. nov. and *G. ocellata* sp. nov. were very similar for *ND2*, in contrast to the phylogenomic data of Ashman et al. (2018). Indeed, no diagnostic nucleotides could be identified for *G. crypta* sp. nov., relative to all other species. Based on phylogenomic data (Ashman et al., 2018), and the existence of clear morphological characters that separate them, we conclude that *G. ocellata* sp. nov. and *G. crypta* sp. nov. are independently evolving lineages, and that the mtDNA signal recovered here is the product of historical introgression between *G. crypta* sp. nov., which is found on the Pilbara mainland, and *G. ocellata* sp. nov., which is found less than 60 km offshore on Barrow Island. Barrow Island has been intermittently connected to the mainland since the Pliocene owing to sea level change, most recently connected approximately 7,000–8,000 years ago (Wilson, 2013). This demonstrates the shortcomings of the single gene approach, and the value of integrating genomic evidence, which inevitably have lower sample sizes per taxon, compared to the large number of specimens that can be sequenced using single gene datasets. Without this integration of phylogenomic, single gene, morphological and geographic datasets, these cryptic species may have remained intractable. Indeed, even more data is required to explore the proposed hypothesis about historical introgression between species in the *G. variegata* species-group.

### Taxonomic conclusions

We base our conclusions on a combination of original mtDNA and morphological analyses, along with the previous genetic studies of Sistrom, Donnellan & Hutchinson (2013) and Ashman et al. (2018). These two studies found evidence for species-level divergences for all new taxa described herein, which we review briefly. Sistrom, Donnellan & Hutchinson (2013) found evidence for multiple cryptic lineages, with *G. versicolor* (their ‘clades IV and V’), *G. pulingka* (‘clade II’) and *G. moritzi* (‘clade I’) described as new species in Hutchinson et al. (2014), with the latter two species having saxicoline habits and appearing as relictual paleoendemic species in the larger *Gehyra* evolutionary tree (Table 1; Ashman et al., 2018). However, Sistrom, Donnellan & Hutchinson (2013)’s ‘clade III’ corresponds to our *G. crypta* sp. nov., for which they had evidence from *ND2*, *H3* and *PRLR* genes.

Ashman et al. (2018) used exon capture to assay diversity across Australian *Gehyra* and found support for all the species described here. However, some notable differences occurred, in that they recovered the sister pairs *G. montium* + *G. crypta* sp. nov. and *G. capensis* sp. nov. + *G. ocellata* sp. nov. In contrast, the mtDNA data had *G. ocellata* sp. nov. nested within *G. crypta* sp. nov., with *G. capensis* sp. nov. as sister to those two species (Fig. 2). Similar occurences of capture of mtDNA through introgression was recently found in northern *Gehyra* (Moritz et al., 2018) and also for arid zone *Uperoleia* frogs (Catullo et al., 2011; Catullo, Doughty & Keogh, 2014). In these cases, nDNA was used as well as mtDNA, enabling detection of such patterns. Lastly, *G. montium* in the mtDNA tree was recovered as just outside of a clade with the species mentioned above, plus *G. pilbara* and the *G. minuta–versicolor* lineage (Fig. 2).

Morphologically, *G. montium* and *G. crypta* sp. nov. most closely resembled each other out of all the species pairs considered here, whereas *G. capensis* sp. nov. and *G. ocellata* sp. nov. also resembled each other by possessing a more spotted pattern on a pinkish or reddish background suggesting more saxicoline habits. Because the latter two species occur in very small areas, the evolution of traits such as colour and pattern may have occurred more rapidly compared to *G. montium* and *G. crypta* sp. nov. that occur over a very wide area in the western arid zone (Fig. 1B) and likely share more generalist habits.

More perplexing was the detection of two species of late Miocene age from the northern Pilbara. *G. incognita* sp. nov. seems to occupy a range among several other arboreal species (Fig. 1), yet the genetic evidence in Ashman et al. (2018) and presented here clearly indicate this is a good biological species. *G. unguiculata* sp. nov. is also from the northern Pilbara and is a relatively old lineage based on the genetic evidence. In this case, however, this species appears to have evolved saxicoline habits and occurs on granites in the northern Pilbara.

In conclusion, we found evidence for the existence of five new species, which we describe below. Although some species were more conspicuous than others, the different lines of evidence strongly indicate they should be regarded as separately evolving entities and hence full species.

### Description of new species

In the descriptions below, and the redescriptions in the Supplemental Material, we outline how the species differ from a generalised description of arid forms from the *G. variegata* group. All the species in this group vary only subtly in body size, shape and morphological characters; therefore, we highlight these differences in the diagnoses, summary tables and comparison sections. Pattern and colouration taken together are more complex and accordingly are more extensively described for each species.

We also include a table of diagnostic loci (Table 3), to be consulted when analysing molecular data to identify species. This will be most useful for the species *G. crypta* sp. nov. and *G. incognita* sp. nov. owing to broad overlap in morphology and geographic distribution with other arid clade species in the *G. variegata* group.

In the ‘comparisons with other species’ sections below, we focus on distinguishing a species from other species that it may co-occur with. For example, in most cases we do not attempt to distinguish western species with central and eastern species such as *G. minuta* or *G. einasleighensis* that occur in entirely different parts of the arid zone. However, if a species is reasonably close to the focal species (e.g. ∼100–200 km), then we include it in the comparisons because at this point in time, distributions are not completely known and we do not want to preclude possible range extensions based on morphology.

We provide common names for the new species below and for previously described species in the Supplemental Materials. We prefer to use ‘Gehyra’ as the common name over ‘dtella’ as the generic name is already available and just as easy or difficult to remember than an additional name fabricated for use as a common name.

*Arid clade of the* G. variegata *group treated herein (Table 1).*

*Diagnosis.* Differs from non-Australian *Gehyra* by lack of extensive webbing between toes III and IV, lack of a fold of skin along the posterior margin of the hindlimb and the presence of wide subcaudal scales. Differs from *G. australis* group species by having smaller body sizes, divided lamellae on the digits and females producing one (not two) eggs per clutch. Differs from the *G. nana* Storr, 1978 clade, *G. punctata* and *G. einasleighensis* by having the inner chin shield separated from the second infralabial.

The five relictual species that fall outside of the *nana* clade and arid clades (Ashman et al., 2018) can be separated from the arid clade of the *G. variegata* group as follows. *G. xenopus* Storr, 1978 and *G. spheniscus* Doughty et al., 2012 differ by possessing a wedge of granules at base of digit, unlike any other *G. variegata* group species. *G. lazelli* and *G. moritzi* share a higher number of chromosomes (2*n* = 44 vs ≤ 42; Hutchinson et al., 2014). *G. pulingka* can be diagnosed by three pairs of chin shields with the third infralabial notched and wavy dark lines and pale spots on the dorsum (Hutchinson et al., 2014).

*Description.* A group of small to moderately large *Gehyra*, body shape dorsoventrally compressed with fine homogeneous rounded scales on dorsum and flattened scales on ventrum, snout relatively short with rounded tip and covered with enlarged rounded scales, eyes large and protruding, ear opening small, wide rostral with paired supranasals and usually two postnasals, nostril in contact with rostral scale, limbs short with claws on digits II–V, claws protruding from dorsal surface of expanded toe pad, divided lamellae on undersides of digits, in males 8–16 pre-cloacal pores in shallow chevron, pores protruding from centre of scale, small cluster of spurs to either side of cloaca, females produce a single egg per clutch, tail cylindrical tapering to a fine point, scales arranged in regular rows on original tails, more irregular on regenerated tails.

***Gehyra capensis* sp. nov.**
urn:lsid:zoobank.org:act:427C7159-A44A-498D-8BDC-1D310E05041C
North West Cape Gehyra
variegataB1 of Ashman et al. (2018)
Figs. 3–5

**Figure 4:**
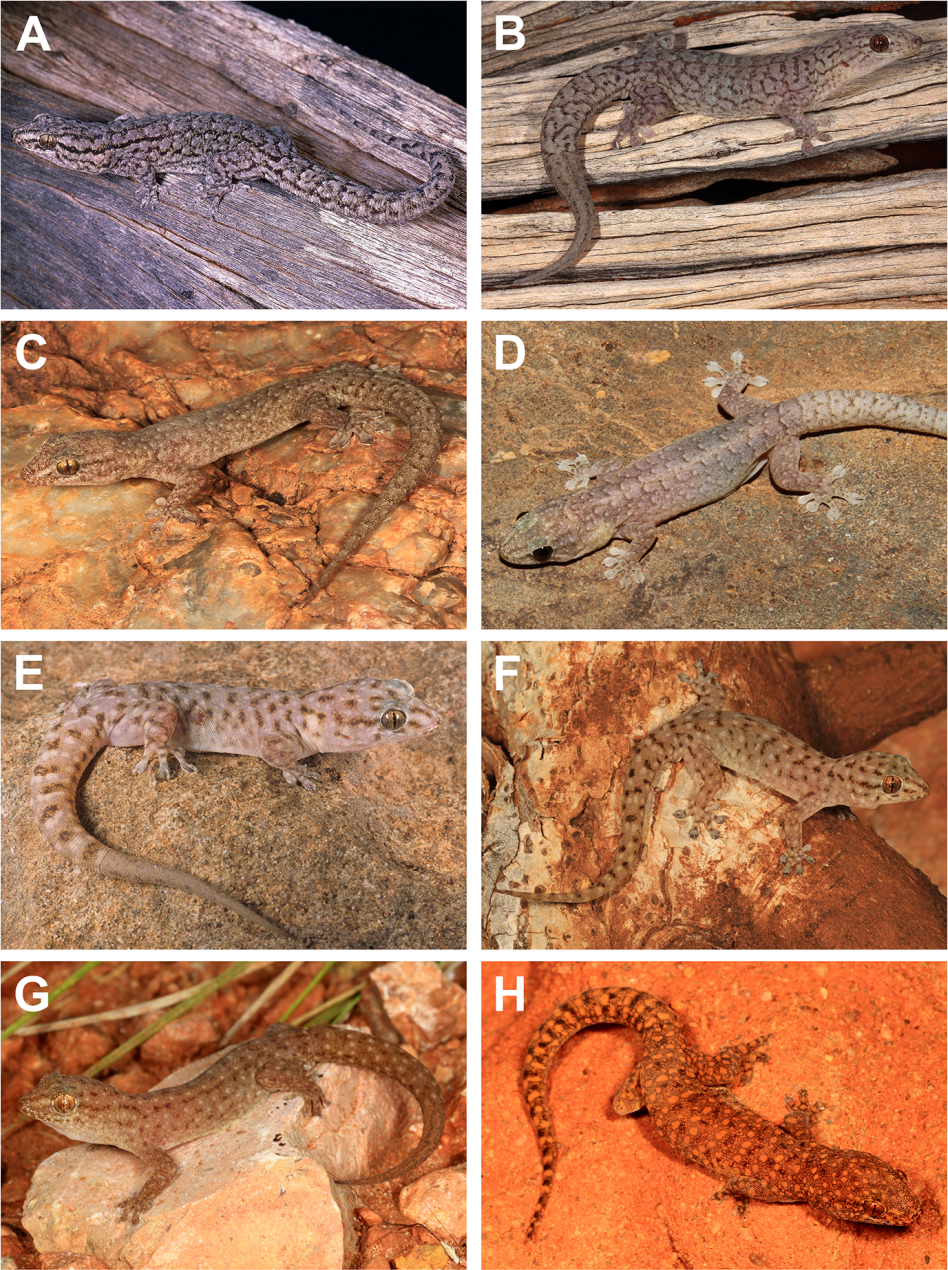
Live images of members of the arid clade of the *G. variegata* group. (A) *Gehyra variegata*, Carey Downs, WA (WAM R119207; photo credit—B. Maryan); (B) *G. purpurascens*, Ilkurlka, WA (B. Maryan); (C) *G. montium*, Skull Springs, WA (WAM R175332; R.J. Ellis); (D) *G. montium*, Port Hedland, WA (WAM R174324; P. Doughty); (E) *G. capensis* sp. nov., Cape Range, WA (B. Maryan); (F) *G. capensis* sp. nov., Cape Range, WA (WAM R174314; R.J. Ellis); (G) *G. ocellata* sp. nov., Barrow Island, WA (R.J. Ellis); (H) *G. pilbara*, Woodie Woodie, WA (R.J. Ellis).

**Figure 5:**
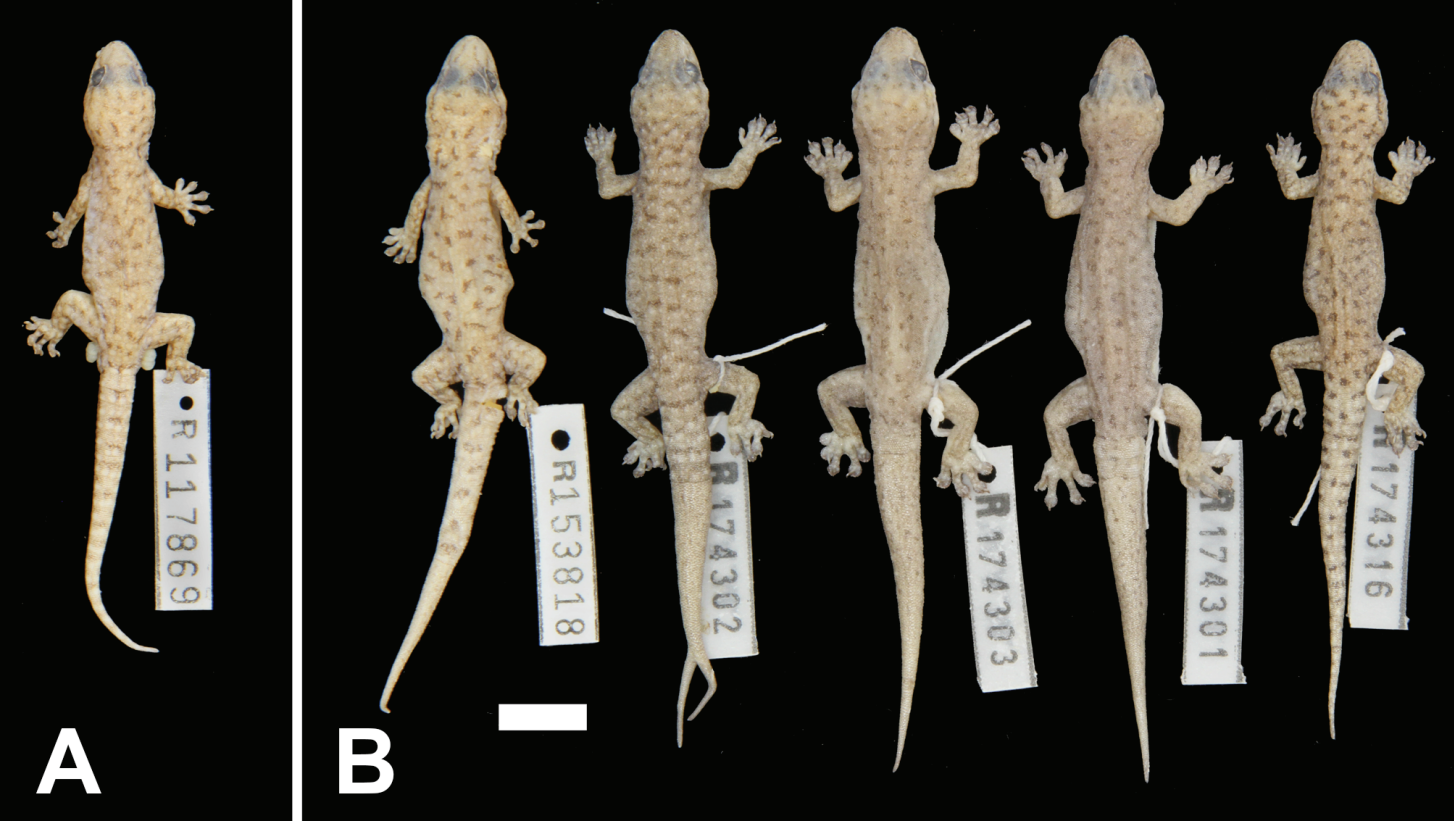
Variation among preserved *Gehyra capensis* sp. nov. specimens. (A) Holotype specimen, (B) paratype specimens. Scale bar = 10 mm. Photo credit—L. Kealley.

*Holotype.* WAM R117869, an adult male collected at Vlaming Head (21°48′S, 114°06′E), WA, on 3 January 2003 by G. Harold and R.J. Teale.

*Paratypes.* WAM R153818 (female), as for holotype; WAM R174300 (male) and WAM R174302 (female), Charles Knife Road, Cape Range National Park (22°05′23″S, 114°00′40″E); WAM R174301 (male), Charles Knife Road, Cape Range National Park (22°05′25″S, 114°00′38″E); WAM R174316 (male), Mandu–Mandu Gorge, Cape Range National Park (22°09′00″S, 113°53′06″E).

*Diagnosis.* A small-bodied (to 46.0 mm SVL) species with a relatively long snout, internarial usually (72%) present, lower postnasal larger than upper, two pairs of chin shields, second infralabial notched by parinfralabial scales, usually six (occasionally seven) subdigital lamellae on the fourth toe and males with 9–12 (mean 10.8) pre-cloacal pores. Background colour pinkish-grey with dark brown irregularly shaped spots or bars with numerous smaller pale white spots not in contact with dark markings, canthal, loreal and temporal stripes on head present (no post-orbital stripes) and ventrum not stippled. Genetically diagnosed from other arid clade members by the *ND2* sites in Table 3.

*Description.* Morphology as for ‘arid clade of the *G. variegata* group treated herein’ description above, with differences outlined in the diagnosis above, Table 2 and Table S2.

*Colour and pattern.* In life, light pinkish-grey brown background colour. Upper surfaces with scattered dark brown irregular spots or short bars and smaller and more numerous pale white spots; the dark and pale markings scattered independently, occasionally in contact. Dark brown canthal and loreal stripes present, temporal stripe continuing posteriorly through the eye to as far as back of head. Tail the same as for dorsum. In preservative, background colour yellowish-grey to dark grey. Dark and pale markings discernible but with loss of contrast. Ventral surfaces pale off-white to cream, little to no stippling.

*Distribution.* Restricted to the North West Cape of WA (Fig. 1B).

*Habitat and ecology.* Recorded from spinifex and low shrubs on limestone rocks. Also encountered under logs and sheets of tin on the ground, and on human-made structures indicating a penchant for climbing behaviour.

*Etymology.* The specific name refers to the North West Cape of WA to which this species is restricted.

*Comparisons with other species.* Based on location, this species could only be confused with *G. variegata* which also occurs on the North West Cape. The two species differ by *G. capensis* sp. nov. attaining a smaller body size (to a maximum SVL of 46.0 vs 52.5 mm), lower postnasal larger than upper (vs equal), pinkish-grey background colour with dark irregular spots or bars and numerous small pale spots not in contact (vs greyish-brown with dark network with pale spots to posterior edges), no post-orbital stripes (vs present) and ventrum without stippling (vs heavy).

*G. capensis* sp. nov. has a similar morphology to the relatively closely related *G. ocellata* sp. nov. on nearby islands to the north and *G. pilbara* on the mainland, but differs by possession of a longer snout with well-defined canthal, loreal and temporal head stripes (vs poorly defined). It differs from *G. crypta* sp. nov. in possessing spots without reticulations (vs short transverse bars often with a dark reticulum) and no stippling on the ventrum (vs medium to dense).

*Remarks.* Specimens of this species had been variously assigned to *G. punctata*, *G. pilbara* or *G. variegata* as this taxon has an intermediate morphology. The first two taxa do not occur on the North West Cape, whereas there are records of *G. variegata* from the low-lying and southern areas on the peninsula. The pattern within *Gehyra* for saxicoline species to possess spots rather than lines or networks suggests *G. capensis* sp. nov. occupies the more rocky habitats of the North West Cape, whereas *G. variegata* maintains its preferences for trees and shrubs.

The North West Cape was previously believed to possess only one endemic reptile species (*Lerista allochira* Kendrick, 1989; Kendrick, 1993), but *G. capensis* sp. nov. is the fourth new gecko species to be described since 2007, joining *Diplodactylus capensis* Doughty, Adams & Oliver, 2007, *Delma tealei* Maryan, Aplin & Adams, 2007 and *Crenadactylus tuberculatus* Doughty, Ellis & Oliver, 2016. The North West Cape is therefore of high conservation value for lizards owing to the relatively large number of endemic species in a small area.

***Gehyra crypta* sp. nov.**
urn:lsid:zoobank.org:act:D700BCA7-ADC6-4B4D-B980-F8B2667BB0DE
Western Cryptic Gehyra
Clade 3 or III of Sistrom, Donnellan & Hutchinson (2013)
variegataB3 of Ashman et al. (2018)
Fig. 6

**Figure 6:**
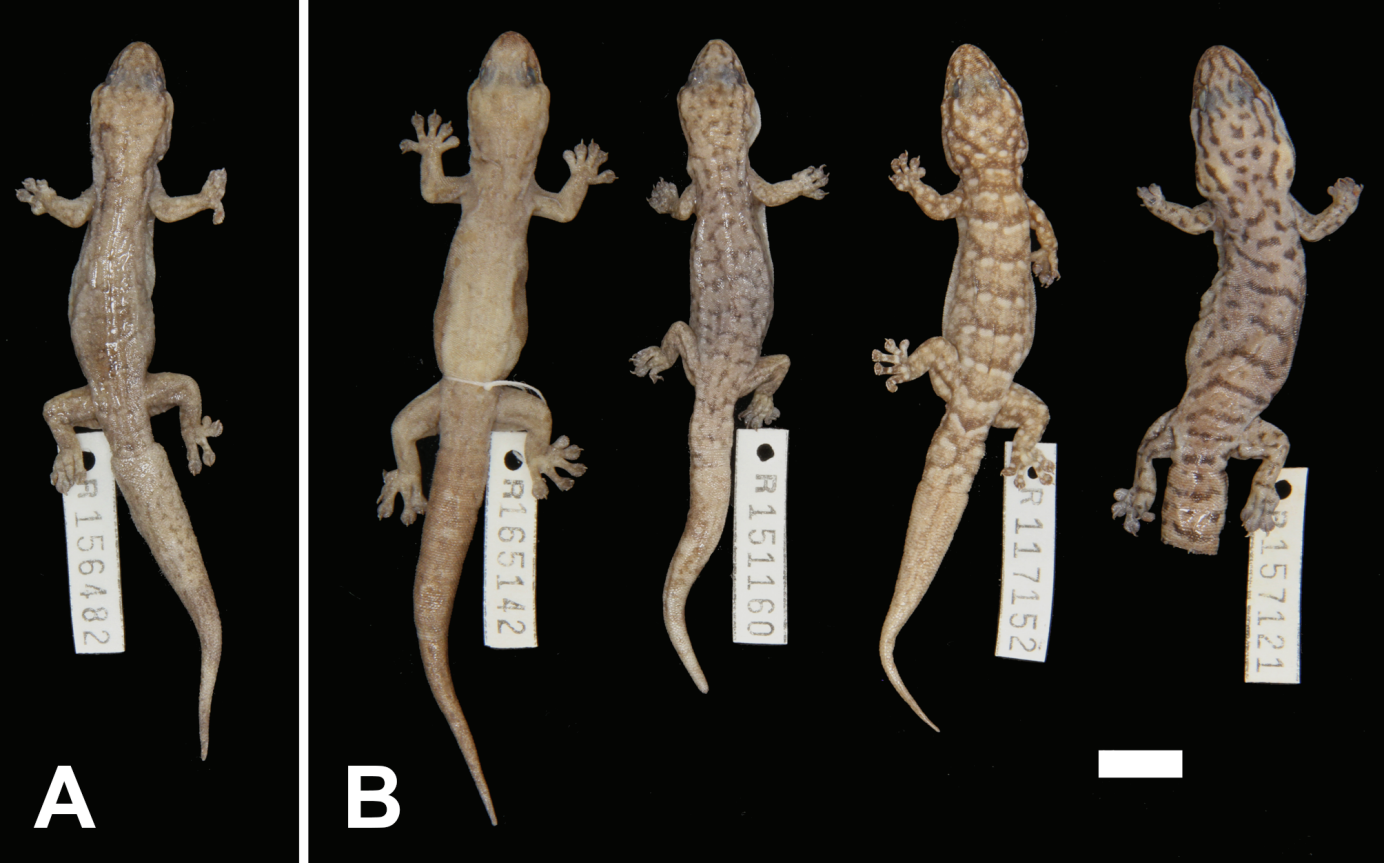
Variation among preserved *Gehyra crypta* sp. nov. specimens. (A) Holotype specimen, (B) paratype specimens. Scale bar = 10 mm. Photo credit—L. Kealley.

*Holotype.* WAM R156482, an adult male collected at Upper Marillana Creek (22°41′S, 118°57′E), WA, on 15 April 2005 by M. Ladyman and colleagues.

*Paratypes.* WAM R111672 (male), one km north–north-west of Mount Bruce (22°34′59″S, 118°27′25″E); WAM R117152 (female), Dead Horse Rocks, 6.5 km north of Menzies (29°22′S, 121°17′E); WAM R151160 (female), Tom Price (22°29′22″S, 117°41′29″E); WAM R157121 (female), West Angelas (23°11′57″S, 118°50′49″E); WAM R165142 (male), 2.1 km north–north-east of Millstream (21°34′38″S, 117°03′44″E).

*Diagnosis.* A moderately sized (to 56.5 mm SVL) species with moderately short snout, internarial usually (80%) present, lower and upper postnasals of similar size, two pairs of chin shields, second or third infralabial notched by parinfralabial scales, usually six or seven (rarely eight) subdigital lamellae on the fourth toe and males with 10–16 (mean 12.4) pre-cloacal pores. In preservative, light grey to dark brown with highly variable pattern: from isolated dark and pale bars to dark network with white spots to patternless, head stripes present but with lower post-orbital stripe at most a spot and ventrum moderately to heavily stippled. Genetically diagnosed from other arid clade members (except *G. ocellata* sp. nov.) by the *ND2* sites in Table 3.

*Description.* Morphology as for ‘arid clade of the *G. variegata* group treated herein’ description above, with differences outlined in diagnosis above, Table 2 and Table S2.

*Colour and pattern.* Pattern highly variable. No known photos in life from genotyped specimens. In preservative, background colour ranges from light grey to dark brown. Markings on dorsum range from: nearly lacking any markings; short light to heavy bars of dark (anterior) and pale (posterior) bars or spots in loose rows along dorsum (either joining to form continuous transverse rows or not in contact with each other); to networks of fine dark lines with scattered white spots in contact with posterior edge of dark lines. Common variations include the lateral edges of the dark markings extending posteriorly to partially encircle pale posterior spots, absence of any pale markings, heavy dark transverse bars with very thin pale lines and individuals with extremely subdued or faded patterns. Clearly defined brown canthal, loreal, temporal and upper post-orbital stripes, lower post-orbital stripe usually absent or at most limited to a dark spot. Original tails with bars formed by anterior dark and posterior pale elements. Ventrum pale off-white to cream with medium density dark stippling.

*Distribution.* Most records are from the southern and western Pilbara, with the northernmost records from the Burrup Peninsula, then inland through Millstream–Chichester National Park through the Hamersley Range to 50 km west of Newman. In the mid-west and WA Goldfields there are scattered genotyped records inland, away from the west coast, through the Gascoyne and Murchison bioregions, with the southernmost records from 150 km north of Kalgoorlie and the easternmost record near Laverton (see Fig. 1B).

*Habitat and ecology.* Possibly generalist habits. Collected from mulga woodlands and acacia shrubs, from under logs, granite and tin on hard soils. Also observed climbing on vertical surfaces of human-made structures and sheltering under bark on trees.

*Etymology.* The species epithet is derived from the Greek *kruptos*, meaning ‘hidden.’ The name alludes to this species similarity to other species in the arid clade of the *G. variegata* group. Used as an adjective.

*Comparisons with other species. G. crypta* sp. nov. is one of the most difficult species to diagnose from others in the group. The range of variation in characters and patterns overlaps with several other species, making identification particularly difficult where more than one species occurs in the same area. However, some species can be eliminated through a combination of characters.

Based on the dorsal and ventral patterning, *G. crypta* sp. nov. either possesses a dark network, isolated dark and pale bars or a plain greyish-brown dorsum which distinguishes it from *G. pilbara*, *G. capensis* sp. nov. and *G. ocellata* sp. nov., as these species have pinkish to reddish colouration. It also has moderate to dense stippling on the ventrum, which also differs from the relatively plain ventrum of these species. As for other species, it further differs from *G. pilbara* by not possessing a short snout.

This species differs from *G. purpurascens* by possessing a smaller body size yet with more numerous pre-cloacal pores in males (10–16 vs 8–11). For the remaining species, *G. variegata*, *G. montium* and *G. incognita* sp. nov. possess well-defined head stripes, whereas *G. crypta* sp. nov. has no lower post-orbital stripe or only a spot. In addition, *G. variegata* and *G. montium* have up to three pairs of chin shields, therefore if a specimen has three chin shields, this would rule out them belonging to *G. crypta* sp. nov. However, these characters are somewhat variable, and so using morphology alone may result in only narrowing down the identification to one of several similar-looking species.

We surmise that juveniles in areas of overlap among species will be nearly impossible to identify confidently with morphology alone. For all such individuals we recommend taking a tissue sample and obtaining diagnostic sequences (Table 3).

*Remarks. Gehyra crypta* sp. nov. was recovered as an independently evolving lineage by both Sistrom, Donnellan & Hutchinson (2013) and Ashman et al. (2018). There was relatively clear separation in the *ND2* gene sequenced here as well, but morphology provided few useful diagnostic characters owing to highly variable morphological traits with wide overlap of character values. Hence, the table of diagnostic *ND2* sites can be used for determining species identification when *G. crypta* sp. nov. overlaps the distribution of other species from the arid clade, and especially its sister species, *G. montium*. Further tests of gene flow between the two species would be of value, as they come into contact in the south-eastern Hamersley Ranges in the Pilbara and possibly further south in the northern Goldfields. These species appear to be recently diverged, which likely explains their similar morphology. Further sequencing of photographed specimens from the range of *G. crypta* sp. nov. may result in live photos that will be useful to assess whether there are diagnostic characters in the pattern and colouration.

***Gehyra ocellata* sp. nov.**
urn:lsid:zoobank.org:act:01CF492A-BF9D-4159-9A78-2D6A51D48AA4
Pilbara Island Gehyra
variegataB2 of Ashman et al. (2018)
Figs. 4, 7

**Figure 7:**
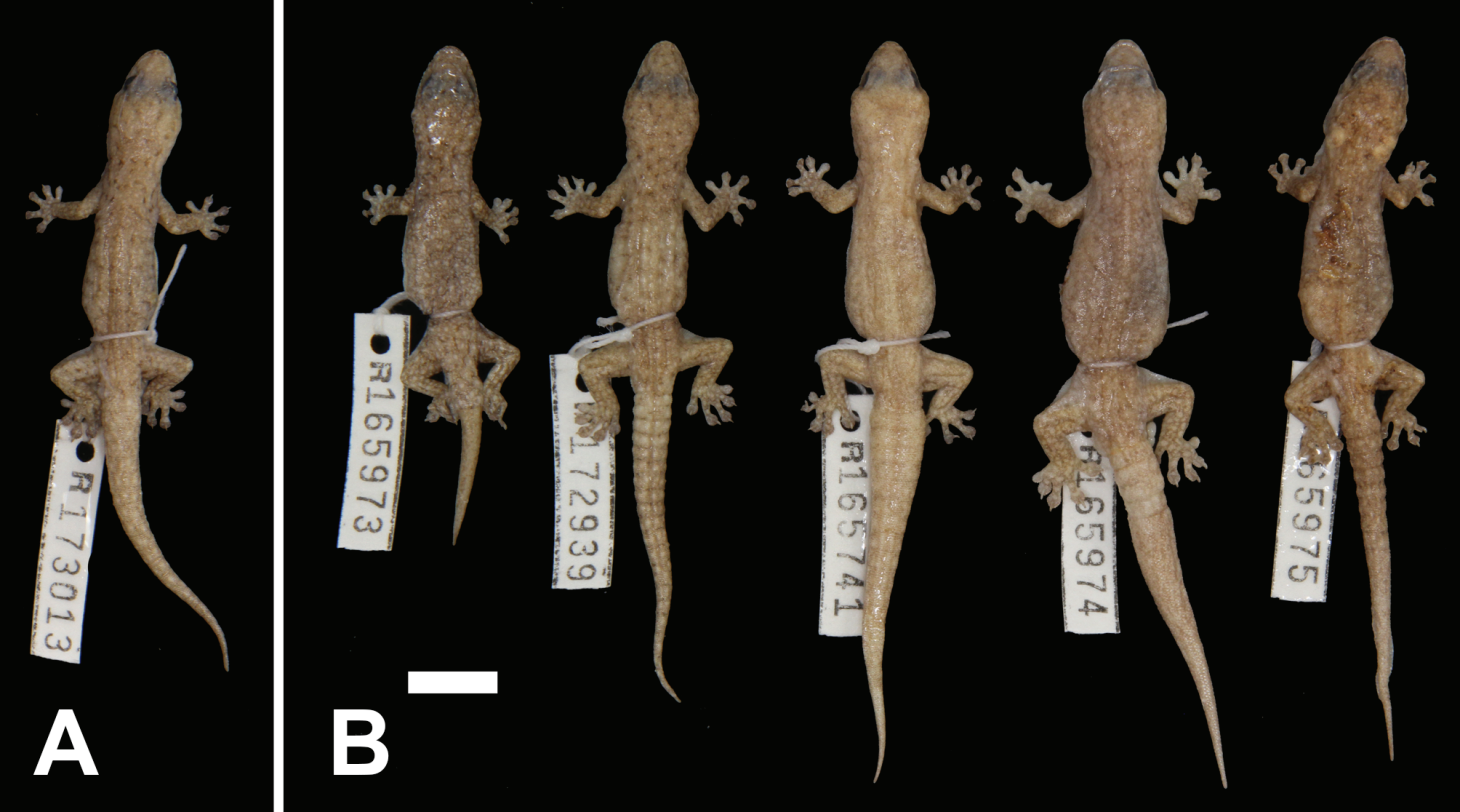
Variation among preserved *Gehyra ocellata* sp. nov. specimens. (A) Holotype specimen, (B) paratype specimens. Scale bar = 10 mm. Photo credit—L. Kealley.

*Holotype.* WAM R173013, an adult male collected at Barrow Island settlement (20°47′46″S, 115°25′52″E), WA, on 11 December 2012 by S. Schmidt (Biota Environmental Sciences).

*Paratypes.* WAM R165971 (male), Trimouille Island (20°23′12″S, 115°33′04″E); WAM R165973 (female), Hermite Island (20°25′22″S, 115°31′54″E); WAM R165974 (male), Hermite Island (20°25′22″S, 115°31′54″E); WAM R165975 (female), Hermite Island (20°25′22″S, 115°31′54″E); WAM R172939 (male), WAPET Barge Landing, Barrow Island (20°43′27″S, 115°28′19″E).

*Diagnosis.* A small-bodied (to 49.0 mm SVL) species with moderately short snout, internarial absent or present, lower postnasal larger than upper, two pairs of chin shields, second infralabial notched by parinfralabial scales, usually six (occasionally seven) subdigital lamellae on the fourth toe and males with 10–12 (mean 11.1) pre-cloacal pores. Background colour light to medium reddish-brown with numerous pale spots with fewer brown irregular markings, head stripes poorly defined or absent and ventrum with little or no stippling. Genetically diagnosed from other arid clade members (except *G. crypta* sp. nov.) by the *ND2* sites in Table 3.

*Description.* Morphology as for ‘arid clade of the *G. variegata* group treated herein’ description above, with differences outlined in diagnosis above, Table 2 and Table S2.

*Colour and pattern.* In life, background colour a light to medium reddish-brown with numerous scattered small pale white spots, with relatively few diffuse fine brown irregular markings among the pale spots, occasionally tending to form a loose network. Crown and limbs as for dorsum, but with fewer dark spots. Brown canthal stripe poorly defined, loreal, temporal and lower post-orbital stripes usually absent but with occasionally a weakly defined upper post-orbital stripe. Original tails as for dorsum, with alternating dark and light spots forming diffuse bands along tail. In preservative, background colour a yellowish tan to light brown. The markings are as for life, but with less contrast. Ventral surfaces pale yellow with little to no dark stippling.

*Distribution.* Restricted to islands off the Pilbara coast near Karratha, including Barrow, Varanus, Trimouille and Hermite (Fig. 1B).

*Habitat and ecology.* Observed to inhabit termite mounds (P. Kendrick, R. J. Teale, 2018, personal communication). Otherwise poorly known, as most records are associated with oil and gas buildings and structures. Several records mention ‘under limestone slab.’

*Etymology.* The species epithet *ocellata* (New Latin) refers to the spotted appearance of this species. Used as an adjective.

*Comparisons with other species.* This species only co-occurs with *G. variegata* on Barrow and surrounding islands. It can be distinguished from *G. variegata*, *G. crypta* sp. nov. and *G. incognita* sp. nov. by possessing a reddish colouration with numerous pale spots with dark brown irregular markings. It is distinguished from *G. pilbara* by the elongate snout and lower postnasal only slightly enlarged relative to upper. It differs from *G. capensis* sp. nov. by having a shorter snout, a more reddish background colour and poorly defined head stripes.

The genetic analyses revealed complete introgression of the mtDNA gene *ND2* into *G. crypta* sp. nov. A similar scenario was recently documented for northern *Gehyra* of the *nana* clade of the *variegata* group (Moritz et al., 2018), demonstrating the possibility of this happening in other *Gehyra* groups. Although indistinguishable on the mtDNA evidence, however, these two species differ in pattern and colouration.

*Remarks.* This species had been previously identified as *G. pilbara* owing to the reddish colouration and pattern of spots. The evolution of a reddish hue and spotted appearance is more often associated with an ecological shift to rocks (e.g. *G. punctata* and *G. nana* clade species), suggesting more saxicoline habits as opposed to the sympatric *G. variegata* on these Pilbara islands which would likely maintain arboreal habits. This situation is also observed for the North West Cape for *G. capensis* sp. nov. and *G. variegata* and may represent a parallel case of ecological divergence in sympatry with only two *Gehyra* species occurring in a small area.

***Gehyra incognita* sp. nov.**
urn:lsid:zoobank.org:act:27959BD3-99C8-4CB9-84C3-7F01979361DD
Northern Pilbara Cryptic Gehyra
variegataC2 of Ashman et al. (2018)
Fig. 8

**Figure 8:**
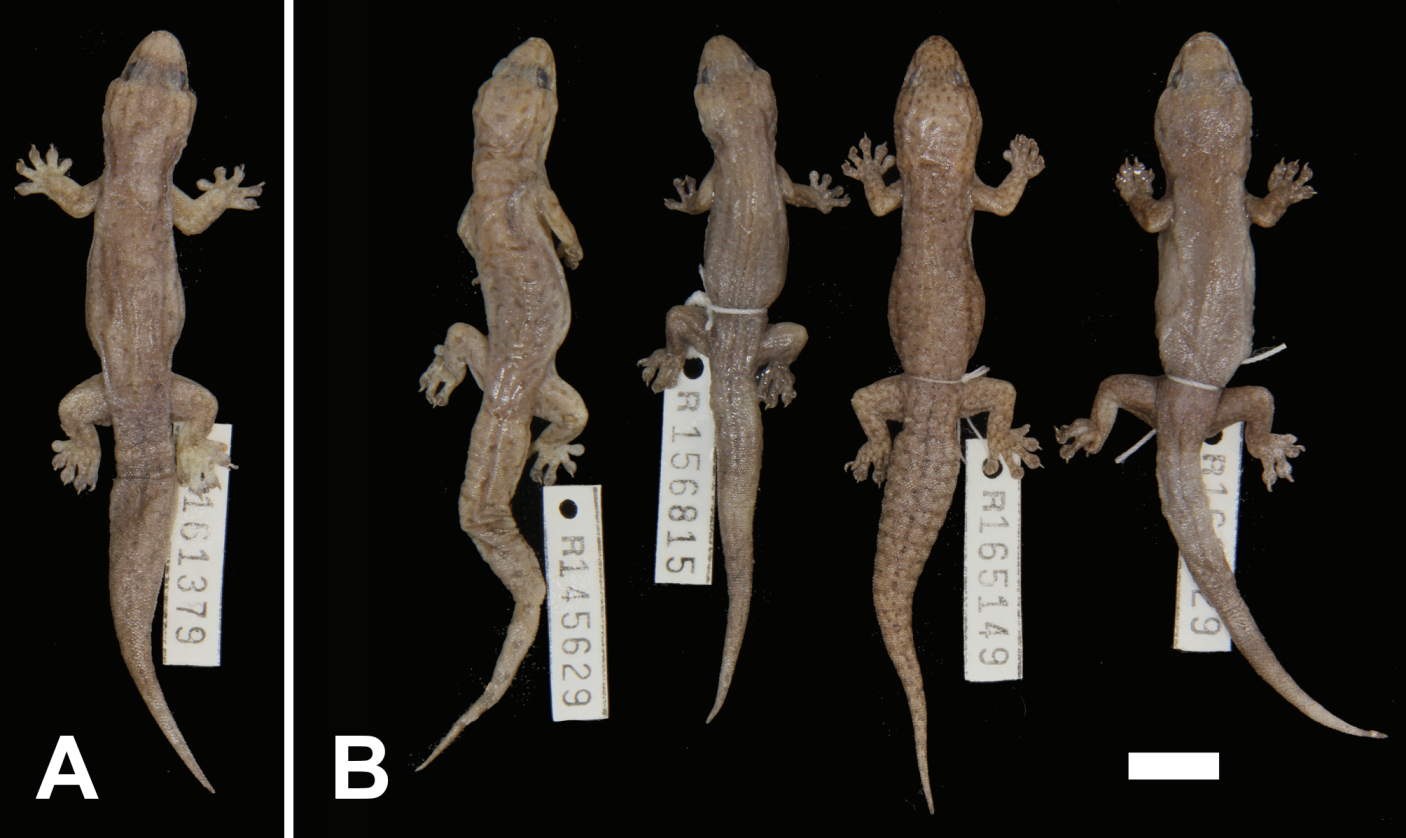
Variation among preserved *Gehyra incognita* sp. nov. specimens. (A) Holotype specimen, (B) paratype specimens. Scale bar = 10 mm. Photo credit—L. Kealley.

*Holotype.* WAM R161379, an adult male collected 38 km north–north-west of Marble Bar, site PHYC06 of the Pilbara Biodiversity Survey (20°50′07″S, 119°40′18″E), WA, on 26 September 2005 by A.H. Burbidge and C. Stevenson.

*Paratypes.* WAM R145629 (male), 18 km south of Port Hedland (20°28′12″S, 118°35′24″E); WAM R156815 (female), Port Hedland area (20°22′49″S, 118°41′52″E); WAM R159848 (male), 12.5 km south of Whim Creek Hotel (20°56′59″S, 117°50′59″E); WAM R165149 (female), five km north–north-east of Python Pool (21°18′37″S, 117°16′34″E); WAM R166629 (female), Mons Cupri Mine (20°51′59″S, 117°49′19″E).

*Diagnosis.* A moderately sized (to 52.0 mm SVL) species with moderately short snout, internarial present or absent, lower postnasal larger or equal to upper, two pairs of chin shields, second infralabial notched by parinfralabial scales, usually six (rarely five or seven) subdigital lamellae on the fourth toe and males with 10–16 (mean 12.0) pre-cloacal pores. In preservative, background colour medium grey to dark brown with poorly contrasting pattern of small dark and pale spots occasionally forming bars or networks, well-defined head stripes and ventrum heavily stippled. Genetically diagnosed from other arid clade members by the *ND2* sites in Table 3.

*Description.* Morphology as for ‘arid clade of the *G. variegata* group treated herein’ description above, with differences outlined in the diagnosis above, Table 2 and Table S2.

*Colour and pattern.* No known photos in life. In preservative, uniform medium grey to dark brown background colour. Markings in this species are usually poorly contrasting with the background colour. Numerous fine dark brown to black spots and smaller pale white spots over dorsal surfaces. In some specimens, short brown transverse bars are present, and more rarely a network, with pale markings posterior to dark markings. Dark brown canthal, loreal, temporal and upper and lower post-orbital stripes present. Original tails as for dorsum. Ventral surfaces off-white to cream with relatively dense stippling.

*Distribution.* Most records are from genotyped individuals that occur near the Pilbara coast and have been collected along the Great Northern Highway. From 40 km east of Roebourne to Whim Creek and to Port Hedland area. Three inland isolated locations: Millstream–Chichester National Park, Woodstock–Abydos Protected Reserve (formerly Station; with several specimens genotyped) and from 40 km north of Marble Bar (see Fig. 1C).

*Habitat and ecology.* Likely arboreal. Habitat notes for quadrats that used pitfall traps for the Pilbara Biodiversity Survey (McKenzie, van Leeuwen & Pinder, 2009) mention tall acacia shrubs over *Triodia*, and substrates that included floodplain, clayey or silty sand and red sandy loam. The only other habitat notes from collectors are two records from *Triodia* plains, with no mention of rocky habitats.

*Etymology.* The specific name is derived from the Latin *incognitus* meaning ‘unknown,’ in reference to the heretofore complete ignorance of this species’ existence prior to genetic analyses. Used as an adjective.

*Comparisons with other species.* This species is also one of the more difficult in the group to identify owing to its relatively plain yet variable appearance. It differs from *G. pilbara* and *G. ocellata* sp. nov. by possessing a medium to dark grey–brown background colour (vs reddish-orange). It differs from the adjacent northern Pilbara species *G. unguiculata* sp. nov. by having a darker background colour with scattered small dark and pale spots and head stripes (vs lighter dorsal colouration with crescent-shaped markings and poorly defined head stripes).

*Gehyra incognita* sp. nov. differs from the similar-looking *G. purpurascens* (which it was often identified in the field as) by smaller maximum body size (52 vs 67 mm), fewer lamellae (6 vs 7–8), two (vs 2–3) chin shields, second (vs second or third) infralabial notched and ventrum heavily (vs little to moderate) stippled. The two species are entirely allopatric (Fig. 1C).

It differs from *G. montium* and *G. crypta* sp. nov. in that the lower postnasal is usually larger than the upper (vs approximately equal in size), two (vs 2–3) chin shields, second (vs second or third) infralabial notched. Further differs from *G. crypta* sp. nov. in having well-defined head stripes (vs lower post-orbital stripe absent or reduced to a spot) and six subdigital lamellae (vs 6–8). From the similar *G. variegata*, it differs in having two pairs of chin shields (vs 2–3) and lower postnasal greater than upper (vs equal).

As the distribution of this species overlaps or abuts several others in the northern Pilbara, we recommend sequencing diagnostic loci to resolve species identity. As there are no known photos in life, such an exercise could yield live images of this species which would be valuable to assist with identification in the field.

*Remarks.* Along with *G. crypta* sp. nov., *G. incognita* sp. nov. is one of the most difficult species in the arid clade to diagnose. There are no known photographs in life, and it possess a darker background colouration which weakens the contrast of the markings. It is most similar to *G. variegata* and *G. purpurascens*, although so far as we know these species do not co-occur together with *G. incognita* sp. nov. in the northern Pilbara. The antiquity of this lineage (late Miocene; Ashman et al., 2018), distribution in the northern Pilbara and close resemblance to other arid clade species are all intriguing. Indeed, it is difficult to imagine detecting the existence of this species without genetic data at all. Photos in life and observations of its behaviour and ecology are warranted to better understand this enigmatic species.

***Gehyra unguiculata* sp. nov.**
urn:lsid:zoobank.org:act:DF3839D0-3AA2-40E6-ACFEF5F47F74EC8B
Crescent-marked Pilbara Gehyra
variegataC1 of Ashman et al. (2018)
Fig. 9

**Figure 9:**
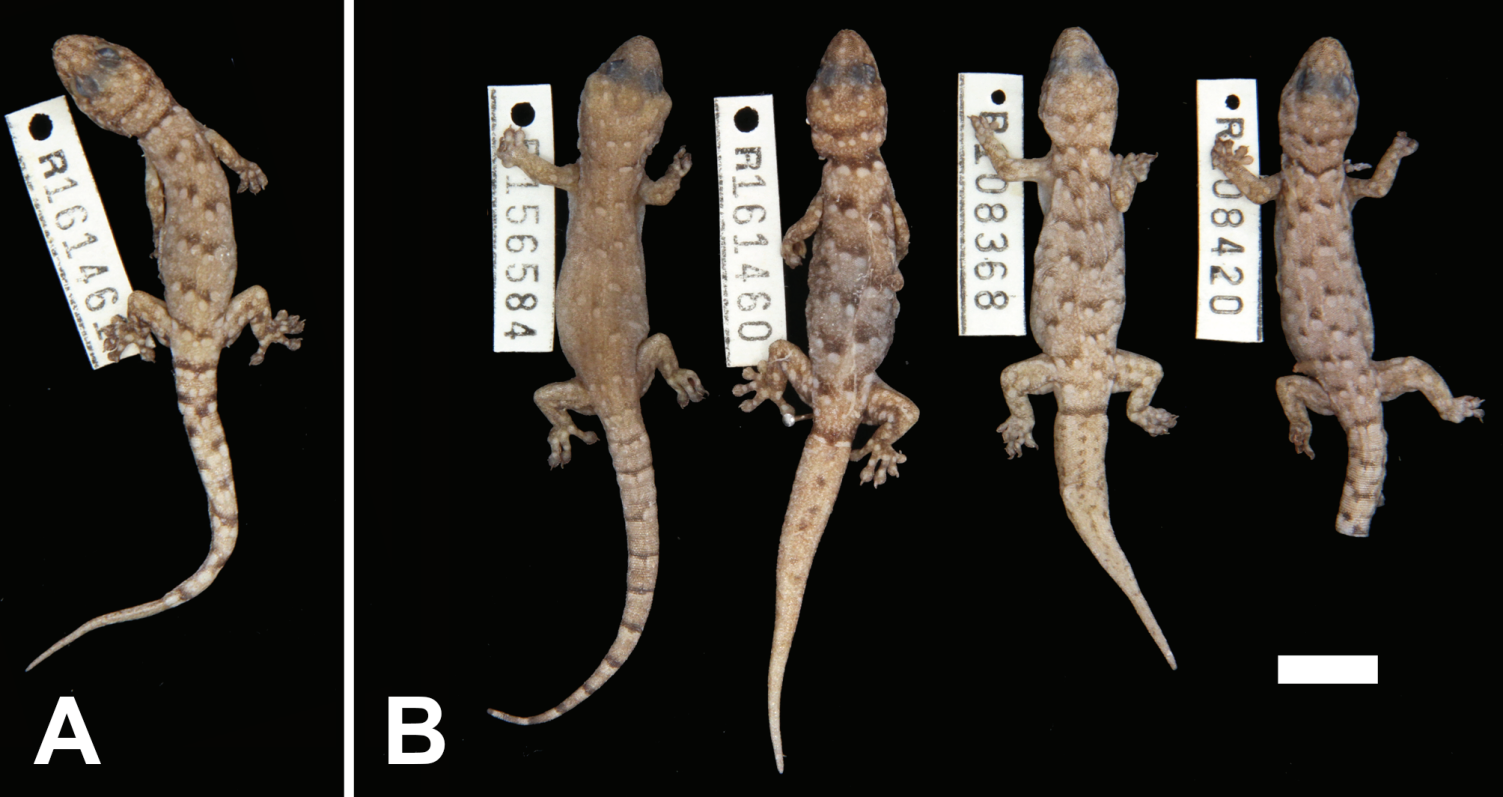
Variation among preserved *Gehyra unguiculata* sp. nov. specimens. (A) Holotype specimen, (B) paratype specimens. Scale bar = 10 mm. Photo credit—L. Kealley.

*Holotype.* WAM R161461, an adult male collected 47.5 km east–south-east of Goldsworthy, site PHYC03 of the Pilbara Biodiversity Survey (20°25′41″S, 119°58′10″E), WA, on 1 October 2005 by A.H. Burbidge and C. Stevenson.

*Paratypes.* WAM R108368 (male), Sunrise Hill (20°27′39″S, 120°02′54″E); WAM R108420 (male), Nimingarra (20°26′11″S, 120°00′39″E); WAM R156584 (male), Cundaline Gap (20°33′28″S, 120°10′55″E); WAM R161459 and WAM R161460 (females), as for holotype.

*Diagnosis.* A small-bodied (to 39.0 mm SVL) species with moderately short snout, internarial present (67%) or absent (33%), lower postnasal larger or equal to upper, two pairs of chin shields, second infralabial notched by parinfralabial scales, usually six (occasionally seven) subdigital lamellae on the fourth toe and males with 11–13 (mean 12.3) pre-cloacal pores. In preservative, background colour light tan to medium brown with crescent-shaped dark brown bars with pale spots posteriorly, crown with pale white spots, usually poorly defined head stripes and ventrum with only light stippling towards lateral edges. Genetically diagnosed from other arid clade members by the *ND2* sites presented in Table 3.

*Description.* Morphology as for ‘arid clade of the *G. variegata* group treated herein’ description above, with differences outlined in the diagnosis above, Table 2 and Table S2.

*Colour and pattern.* No known photos in life. In preservative, light tan to medium brown background colour. Markings on dorsum with dark brown anterior bars in contact with 1–3 pale white circular or oblong spots; anterior dark bar tending to curve around anterior portion of pale spot or spots; markings in seven or eight rows from nape to hindlimbs and from two to three spots along the width of the body; markings on nape usually forming continuous transverse lines. Dark brown canthal, loreal and temporal stripes are poorly defined to absent; on crown only pale white spots. Original tails with dark brown bands or rows of spots (anterior) and pale white rows of spots (posterior) on proximal portion, distal portion with less orderly arrangement. Ventral surfaces pale off-white to cream.

*Distribution.* Only known from two locations 30 km apart in the north-eastern Pilbara near Shay Gap, north of the De Grey River (Fig. 1C).

*Habitat and ecology.* Several specimens were captured in pitfall traps at sites PHYC03 and PHYC07 as part of the Pilbara Biodiversity Survey (McKenzie, van Leeuwen & Pinder, 2009). Descriptions for these sites were of scree on or near granite outcrops or hills of basalt.

*Etymology. Unguiculata* is Latin (diminutive) for fingernail (or claw) and refers to the resemblance of the dorsal pattern elements of this species to small fingernails. Used as an adjective.

*Comparisons with other species.* This small-bodied species possesses a highly distinctive dorsal pattern of crescent-shaped dark and pale markings and ventrum with light stippling on edges but with central area immaculate. From other co-occurring species it differs from *G. pilbara* by possessing a snout of moderate length (vs very short) and brownish dorsal colouration (vs reddish-orange). From *G. purpurascens* it differs by much smaller body size (maximum 39 vs 67 mm), fewer fourth toe lamellae (6 vs 7–8), more numerous pre-cloacal pores (11–13 vs 8–11) and the unique dorsal markings. From the slightly larger species *G. montium* and *G. incognita* sp. nov. it differs by the dorsal pattern and from *G. montium* by having two (vs 2 or 3) chin shields.

*Remarks.* As for *G. incognita* sp. nov., the age of this species (late Miocene; Ashman et al., 2018) and small distribution in the northern Pilbara are intriguing. The habitat from pitfall sites associated with granite outcrops and a dorsal pattern of spots without head stripes suggests a saxicoline ecology. More observations of animals in the field, however, are necessary to test this supposition. The patterning tends to overlap that of *G. punctata*, but the greyish background colouration of *G. unguiculata* sp. nov. is different than the reddish hues in *G. punctata*, possibly as an adaptation to the greyer colour of granite outcrops in the northern Pilbara vs the reddish ironstones that are more common elsewhere in the Pilbara (Pepper, Doughty & Keogh, 2013). The northern Pilbara is less well sampled than the southern Pilbara owing to its less lucrative minerals and thus fewer environmental surveys for fauna. A targeted survey of granitic rocks in the northern Pilbara could reveal this species is more widely distributed than we have documented here.

## Conclusions

The systematics of *Gehyra* is intrinsically difficult owing to their conservative form, ability to shift habitat preferences from trees to rocks (Ashman et al., 2018), rapid evolution of body size (Doughty et al., 2012; Sistrom et al., 2012), a large specimen burden, few photographs in life and colours and pattern lost rapidly in preservative, among others. We generated relatively short sequences of the *ND2* mtDNA gene for over 650 specimens, combining these with previously genotyped specimens and examining hundreds of specimens for morphology. We were able to make progress by combining these data with previous phylogenomic work that included hundreds of nDNA loci and that firmly established the existence of cryptic species, but based on few specimens. This combined approach is especially suited for cryptic species, as a good understanding of the geographical and morphological limits of cryptic species requires examination of many specimens over a wide area. The combined approach we advocate here culminated in the description of five new species, and clarified the distributions of previously described species as well.

Although great progress has been made on this group taxonomically, there is still further work to be done. Owing to the difficulty of describing these new species, it is clear that photographs in life are an essential part of *Gehyra* specimen preparation because they capture the patterning, a key component of the phenotype, and should be routinely taken when collecting them. Genetic samples (e.g. tail tip in ethanol) of *Gehyra* taken in the field are also essential to determine which species occurs in a specific location, especially where two or more cryptic forms overlap. Both these technologies (digital photography and genetic analyses) were not available only several decades ago, and images and tissue samples should be routinely collected by field workers today. Refinement of the diagnoses and descriptions beyond that presented here based on new phenotypic and genetic information are welcome, and may even reveal further cryptic forms within this difficult group.

## Supplemental Information

10.7717/peerj.5334/supp-1 Supplemental Information 1 Brief redescriptions of arid zone *variegata* group species that have been previously described. Below we briefly redescribe species within the arid clade of the *variegata* group. Tables 2 and S1 provide further information. The redescriptions are based on the specimens we examined in Table S2, and also the previous work of Hutchinson et al. (2014).

10.7717/peerj.5334/supp-2 Supplemental Information 2 Table S1. Morphological summary. Covers all species treated herein, divided by sex (M or F). Sample sizes are provided after the species name and sex. Abbreviations and definitions of characters: SVL (snout-vent length)–from tip of snout to anterior edge of cloaca; TailL (tail length)–from posterior edge of cloaca to tip of tail, original tails only; HeadL (head length)–measured obliquely from tip of snout to retroarticular process at edge of jaw; HeadW (head width)–measured at widest part of head between eyes and ears; HeadD (head depth)–measured between eyes and ears; OrbitL (orbit length)–measured horizontally at widest point; EyeNarL (eye to naris length)–measured obliquely from anterior corner of eye to centre of nostril; SnoutEyeL (snout to eye length)–measured obliquely from anterior corner of eye to tip of snout; InterOrbL (interorbital length)–from anterior corners of eyes; InterNarL (internarial length)–measured from centre of each nostril; LegL (leg length)–from knee to heel; SupLab (supralabials), InfLab (infralabials)–enlarged scales counted until size reached that of normal background scales; Internarials–number of small scales between enlarged supranasals; 4TLam (fourth toe lamellae)–number of enlarged lamellae on the underside of the fourth toe; Pre-cloacal pores–number of pores anterior to cloaca in males, pore perforating centre of enlarged scales.

10.7717/peerj.5334/supp-3 Supplemental Information 3 Table S2. Specimen information for species treated here. All specimens are from the Western Australian Museum (R prefixes omitted from the registration numbers). ‘Genotyped’ means mtDNA was sequenced, and ‘Morphology’ refers to specimens included in the morphological analyses.

10.7717/peerj.5334/supp-4 Supplemental Information 4 Raw genetic data and GenBank accession numbers.

10.7717/peerj.5334/supp-5 Supplemental Information 5

## Abbreviations

MNHN: Muséum Nationale d’Histoire Naturelle, Paris
NT: Northern Territory
NTM: NT Museum & Art Gallery
SA: South Australia
SAMA: SA Museum

## Additional Information and Declarations

### Field Study Permissions

The following information was supplied relating to field study approvals (i.e. approving body and any reference numbers): Field collections were approved by the Department of Parks & Wildlife (WA) (08-000214-2).

### DNA Deposition

The following information was supplied regarding the deposition of DNA sequences: Sequences have been deposited on GenBank and are also accessible from the FASTA file in the Supplemental Materials.

### Data Availability

The following information was supplied regarding data availability: Morphobank: P3186, http://morphobank.org/permalink/?P3186.

### New Species Registration

The following information was supplied regarding the registration of a newly described species: Publication LSID: urn:lsid:zoobank.org:pub:D3B2FF0E-C0D7-4E52-B3D5-CC37A60DC8B6; Gehyra capensis: urn:lsid:zoobank.org:act:427C7159-A44A-498D-8BDC-1D310E05041C; Gehyra crypta: urn:lsid:zoobank.org:act:D700BCA7-ADC6-4B4D-B980-F8B2667BB0DE; Gehyra ocellata: urn:lsid:zoobank.org:act:01CF492A-BF9D-4159-9A78-2D6A51D48AA4; Gehyra incognita: urn:lsid:zoobank.org:act:27959BD3-99C8-4CB9-84C3-7F01979361DD; Gehyra unguiculata: urn:lsid:zoobank.org:act:DF3839D0-3AA2-40E6-ACFE-F5F47F74EC8B.
