# Supplementary material for: Conspicuously concealed: revision of the arid clade of the Gehyra variegata (Gekkonidae) group in Western Australia using an integrative molecular and morphological approach, with the description of five cryptic species
Source: PeerJ. 2018 Jul 19;6:e5334. doi: 10.7717/peerj.5334 (PMC6054870; doi:10.7717/peerj.5334)
Supplement: Supplemental Information 1 — Below we briefly redescribe species within the arid clade of the variegata group. Tables 2 and S1 provide further information. The redescriptions are based on the specimens we examined in Table S2, and also the previous work of Hutchinson et al. (2014). [file peerj-06-5334-s001.docx]

**Supplemental section: Brief redescriptions of previously-described species.** Below we briefly redescribe species within the arid clade of the *variegata* group. Tables 2 and S1 provide further information. The redescriptions are based on the specimens we examined in Table S2, and also the previous work of Hutchinson et al. (2014).

***Gehyra variegata* (Duméril & Bibron, 1836)**

Western variegated gehyras

*Lectotype.* MNHN 2295, Shark Bay, Western Australia.

*Diagnosis.* A moderately-sized (to 52.5 mm SVL) species with moderately short snout, internarial present or absent, upper and lower postnasals approximately equal in size, 2 or 3 pairs of chin shields, 2^nd^ infralabial notched by parinfralabial scales, usually 6 (occasionally 7 or 8) subdigital lamellae on the 4^th^ toe and males with 10–15 (mean 11.5) pre-cloacal pores. Background colour a light to dark grey-brown usually overlain with a dark network with pale posterior edges or spots, well-defined head stripes and heavily stippled ventrum.

*Description.* See Hutchinson *et al.* (2014), Table 2 and Table S1 for the morphology and character redescriptions.

*Colour and pattern.* In life, light to dark grey-brown background colour; short black to brown bars scattered over dorsum, usually in contact with other bars forming a network or ‘vaguely ladder-like pattern’ (Hutchinson *et al.*, 2014) if there are dark longitudinal stripes on the dorsum. On the posterior edge of the dark bars a pale white spot or bar. A common variation is nearly plain individuals without markings; less common is a pattern with dark longitudinal lines only (no transverse bars) or pale markings lacking altogether. Populations associated with rocks have larger spots (Hutchinson *et al.*, 2014).

On the snout, dark brown canthal and loreal stripes usually present, loreal stripe continuing posterior to eye as strong temporal stripe that often continues past neck onto dorsum; upper and lower post-orbital stripes usually present. Tail with bars on dorsal surface, whole or broken along length of tail, anterior portion dark and posterior portion pale as for bars on dorsum; lateral surfaces of proximal portion of tail with dark longitudinal lines. Regenerated tails greyish brown with distorted or no pattern.

In preservative, contrast of markings reduced and background colour usually fades to a paler shade of grey-brown. Ventrum pale off-white and heavily stippled.

*Distribution.* Broadly occurs along the south-western edge of the Australian continent (Fig. 1A). From west to east, as follows. In the Pilbara, around Karratha on the coast, inland south of Pannawonica with scattered records near Newman and the eastern Hamersley Range. Along with western coast, including Barrow Island, North West Cape and Abrolhos Islands, generally within 150 km to the coastline. From ~28°S, it occurs widely in the south-west, including the Darling Range, Wheatbelt and Western Goldfields, but is absent from the southern coast from south of Perth to Cape Arid. Extends south-eastwards through to South Australia, to approximately 100 km west of Ceduna.

*Habitat and ecology.* Arboreal, but also associated with rocky areas. It inhabits shrubs and trees, sheltering under bark or in crevices. Also shelters under rocks, fallen logs and other ground cover.

*Etymology.* *variegata* is Latin for variable, in reference to this species’ appearance.

*Comparison with other species.* *Gehyra variegata* can be distinguished from species that are likely to co-occur or be adjacent to it by not possessing reddish colouration (*G.* *pilbara*, *G. ocellata* sp. nov.) and a network of dark lines on the dorsum (*G. pilbara*, *G. montium*, *G. capensis* sp. nov., *G. ocellata* sp. nov., *G. incognita* sp. nov., *G. unguiculata* sp. nov.).

It is most similar to *G. purpurascens*, *G. versicolor* and *G. crypta* sp. nov. We provide some diagnostic characters from morphology that may separate these extremely similar-looking species, however, genetic analysis of a tissue sample may be required if the specimen is collected from a zone of overlap. From *G. purpurascens* it is distinguished by smaller body size, postnasal size similar (vs larger), fewer 4^th^ toe lamellae (mean of 6.3 vs 7.4), fewer pre-cloacal pores (range 10–15 vs 8–11) and more dense stippling on the ventrum. From *G. versicolor*, it is practically indistinguishable morphologically, but postnasals tend to be similar in size (vs larger > upper) and differs by karyotype (2n = 40b vs. 2n = 40a or 38a) and the species replace each other geographically (Hutchinson et al., 2014). *Gehyra crypta* **sp. nov.** is also almost indistinguishable from *G. variegata*. However, the lower post-orbital stripe in *G. variegata* is strong whereas in *G. crypta* sp. nov. it is reduced to at most a spot.

*Remarks.* The name *Gehyra variegata* used to apply to geckos across the Australian arid zone, but owing to the recent genetic work, its range has been considerably reduced. The species *G. crypta* sp. nov., *G. incognita* sp. nov., *G. lazelli*, *G. montium* and *G. versicolor* now apply to former populations of *G. variegata* throughout most of the arid zone, leaving true *G. variegata* to coastal and arid Western Australia and southern South Australia.

***Gehyra versicolor* Hutchinson, Sistrom, Donnellan & Hutchinson, 2014**

Eastern variegated gehyras

*Holotype.* SAMA R51968, 1.9 km SW of Reedy Hole Springs, Flinders Ranges, South Australia (30°15'55"S, 138°49'30"E).

*Diagnosis.* A moderately-sized (to 58.5 mm SVL) species with moderately short snout, internarial present or absent, lower postnasal larger than upper, 2 pairs of chin shields, 2^nd^ infralabial notched by parinfralabial scales, usually 7 subdigital lamellae on the 4^th^ toe and males with 13–15 (mean 14.0) pre-cloacal pores. Background colour a light to dark grey-brown usually overlain with a dark network with pale posterior edges or spots, well-defined head stripes and heavily stippled ventrum. Chromosome arrangement of 2n = 40a or 38a.

*Description.* See Hutchinson et al. (2014).

*Colour and pattern.* As for *G. variegata*, except with lesser contrast (Hutchinson et al., 2014).

*Distribution.* Broadly distributed throughout the eastern arid zone in South Australia, southern portion of the Northern Territory, arid zones of Queensland and New South Wales and the north-western edge of Victoria. Known in Western Australia from a single genotyped specimen (WAM R98103; Fig. 1A).

*Habitat and ecology.* As for *G. variegata*, a largely arboreal generalist, as most ecological observations from eastern Australia would have been of *G. versicolor* (e.g. Bustard, 1968; Bustard, 1969).

*Etymology.* *versicolor* is Latin for variable in colour (Hutchinson et al., 2014).

*Comparison with other species.* Differs from *G. minuta* by possessing larger body size, greyish-brown dorsal colouration with network of dark lines and pale spots, relatively shorter snout, lower postnasal larger, but not excessively so, than upper and up to 3 (vs 2 only) chin shields. Differs from *G. variegata* by chromosome arrangement (2n = 40a or 38a vs 40b; Hutchinson et al., 2014), lower postnasal larger than upper (vs equal) and less contrast in dorsal pattern. Differs from *G. montium* by having a network of dark lines and spots (vs short dark brown bars connected posteriorly with spots). Differs from *G. purpurascens* by smaller maximum body size (58 vs 67 mm SVL), more pre-cloacal pores (13–15 vs 8–11) and lacks purplish colouration in life.

***Gehyra minuta* King, 1982**

Dwarf gehyras

*Holotype.*NTM R9878, 78.5 km south of Renner Springs, Northern Territory.

*Diagnosis.* A diminutive (to 40.0 mm SVL) species with relatively long snout, internarial usually present, lower postnasal much larger than upper, 2 pairs of chin shields, 2^nd^ infralabial notched by parinfralabial scales, 6 or 7 subdigital lamellae on the 4^th^ toe and males with 10–14 (mean 12.0) pre-cloacal pores. Background colour a reddish-brown with irregular dark brown markings (lines or spots) and small pale white spots usually not in contact, canthal, loreal and temporal stripes present but post-orbital stripes absent and ventrum with moderate stippling.

*Description.* See King (1982) for a detailed description, including colour and pattern.

*Colour and pattern.* Background colour a reddish-brown with irregular dark brown markings (lines or spots) and small pale white spots usually not in contact, canthal, loreal and temporal stripes present but post-orbital stripes absent and ventrum with moderate stippling. Tail continues pattern of dorsum, tending to form bands.

*Distribution.* Restricted to rocky ranges in the central portion of the Northern Territory.

*Habitat and ecology.* Saxicoline.

*Etymology.* *minuta* is derived from Latin *minutus*, meaning small.

*Comparison with other species.* Differs from *G. purpurascens* and *G. versicolor* with which it co-occurs by smaller body size, relatively long snout, lower postnasal much larger than upper, at most only 2 chin shields with 2^nd^ infralabial notched (never 3 chin shields or 3^rd^ infralabial notched) and reddish colouration.

***Gehyra pilbara* Mitchell, 1965**

Pilbara or termitaria gehyras

*Holotype.* WAM R20046, from Tambrey Homestead on the Pilbara plateau, Western Australia (21°38'S, 117°36'E).

*Diagnosis.* A moderately-sized (to 51.5 mm SVL) species with shortened snout, internarial present or absent, lower postnasal much larger than upper, 2 pairs of chin shields, 2^nd^ infralabial notched by parinfralabial scales, usually 7 (occasionally 6 or 8) subdigital lamellae on the 4^th^ toe and males with 10–14 (mean 12.5) pre-cloacal pores. Background colour a dull orange to reddish-brown with scattered dark brown spots or bars and more numerous white spots usually not in contact, weak canthal and temporal stripes (no other head stripes) and ventrum with little or no stippling.

*Description.* See Mitchell (1965) for a detailed description, plus Table 2 and Table S1.

*Colour and pattern.* In life, background colour orange to reddish-brown. Fine dark flecks scattered over dorsum creating slightly stippled appearance. Scattered over dorsum are dark brown spots, occasionally forming short bars, and more numerous clearly-defined pale white to yellow spots that extend to head and upper surfaces limbs and that are usually not in contact with the dark markings. Weak canthal stripe light brown, no loreal stripe, dark brown temporal stripe occasionally present but not strongly defined. On tail, continuation of dorsal pattern with dark bars and white spots.

In preservative, dull yellow to light orange to medium brown background colour, with poorer contrast of pale and dark markings. Ventrum pale off-white to cream, with little to no stippling.

*Distribution.* Ranges from the Pilbara region in the western portion of its range, through to the northern part of the western deserts to the central Northern Territory (Fig. 1A). The eastern-most records are from north of the Simpson Desert, approximately 140 km west of the NT-Queensland border (NTM R22786–87).

*Habitat and ecology.* This species has a strong association with termite mounds and likely has an obligate reliance on termites as a food source. Also forages away from mounds at night.

*Etymology.* Named for the Pilbara region from which this species was described by Mitchell (1965). Owing to the marked increase of *Gehyra* species descriptions in the region and this species’ strong habitat preference for termite mounds, we suggest the common name of ‘termitaria gehyras’ (rather than ‘Pilbara gehyras’).

*Comparisons with other species.* *Gehyra pilbara* is the most distinctive of the species within the arid clade of the *variegata* group. It has a characteristically short snout with chin shields of reduced length which are unique in the group, as well as the lower postnasal being much larger than the upper. The background colour is orange to reddish-brown with numerous pale white spots and dark brown spots or bars not in contact, head stripes absent (no loreal or post-orbital stripes) or greatly reduced (canthal and temporal) and ventrum with reduced stippling. This pattern is similar to *G. capensis* sp. nov., but that species is more pinkish-grey and has a relatively elongate snout, and to *G. ocellata* sp. nov. which tends to have more lamellae on the 4^th^ toe (6.9 vs 6.2).

***Gehyra montium* Storr, 1982**

Mountain gehyras

*Holotype.* WAM R31732, Mt Lindsay, South Australia (27°02'S, 129°53'E).

*Diagnosis.* A moderately-sized (to 49.5 mm SVL) species with moderately short snout, internarial almost always (90%) present, lower postnasal larger or equal to upper, 2 or 3 pairs of chin shields, 2^nd^ or 3^rd^ infralabial notched by parinfralabial scales, usually 7 (occasionally 6) subdigital lamellae on the 4^th^ toe and males with 10–15 (mean 12.0) pre-cloacal pores. Background colour greyish-brown with short brown bars in contact with pale white markings, well-defined head stripes and ventrum heavily stippled.

*Description.* See Hutchinson et al. (2014) for a redescription.

*Colour and pattern.* In life, from light grey-brown to pinkish or reddish-brown background colour. Typically short bars of dark brown (anterior) in contact with pale white (posterior) markings in the Pilbara and western deserts. More common in the central ranges is a pattern similar to *G. variegata* with thin dark brown lines forming a network/reticulum over the dorsum (see Hutchinson et al., 2014), and in some individuals only longitudinal stripes present. Posterior edge of dark lines with scattered pale white spots or short bars usually in contact, less frequently pale spots framed by dark lines. Clearly defined canthal, loreal, temporal and upper and lower post-orbital stripes, temporal stripe usually continuing as dorsolateral stripe on dorsum. Tail with irregular dark bars usually bordered posteriorly by pale white spots.

In preservative, background colouration a greyish-brown with markings highly subdued. Ventrum pale off-white to light greyish-brown in heavily stippled specimens.

*Distribution.* Occurs in the western arid zone, from the central ranges in eastern WA and northern SA in the eastern part of the range (where it was originally believed to be confined to until 2013), with isolated records in the western deserts of WA, including as far south as Kalgoorlie and abundant records from the eastern half of the Pilbara (Fig. 1B).

*Habitat and ecology.* A range of rocky and woodland habitat types have been recorded by collectors in the WAM database, indicating generalist habits.

*Etymology.* The species epithet is Latin for ‘of the mountains’ (Storr, 1982). Although not strictly occuring on mountains, we favour the retention of the common name for the simple reason of there being many similar-looking *Gehyra* species that are in need of common names that are easy to remember.

*Comparisons with other species.* *Gehyra montium* shares a similar size and habitus to several other species and occurs on both rocks and trees, making identification difficult. As with all other species treated here, it differs from *G. pilbara* by possessing a moderately long snout. The character of possessing at least one internarial scale (i.e. separating the supranasals) can help distinguish it from several similar-looking species (*G. variegata*, *G. purpurascens*, *G. versicolor*, *G. crypta* sp. nov., *G. incognita* sp. nov.). This species can possess a pinkish to reddish-brown hue, especially in the central ranges, which can distinguish it from the greyish-brown species listed immediately above. In the Pilbara region, *G. montium* has isolated short dark and pale bars, rather than networks as in the central populations. The heavy stippling on the ventrum of *G. montium* distinguishes it from *G. pilbara*, *G. purpurascens* and *G. unguiculata* sp. nov.

*Remarks.* The distribution of *G. montium* was greatly expanded by Sistrom et al. (2013) when they found many specimens from the Pilbara region that were previously classified as *G. variegata*, *G. punctata* or *G. pilbara*. The former two taxa are known to have harboured several cryptic species, and with *G. montium* having an intermediate appearance, workers would have identified specimens as belong to these variable groups.

***Gehyra purpurascens* Storr, 1982**

Purple gehyras

Holotype. WAM R72660, 3.5 km north-east of Comet Vale, Western Austrlia (29°55'S, 121°08'E).

*Diagnosis.* A large-sized (to 67.0 mm SVL) species with moderately short snout, internarial usually (83%) present, lower postnasal larger or equal to upper, 2 or 3 pairs of chin shields, 2^nd^ or 3^rd^ infralabial notched by parinfralabial scales, usually 7 or 8 (rarely 6) subdigital lamellae on the 4^th^ toe and males with 8–11 (mean 9.7) pre-cloacal pores. Background colour purplish grey to brown with a dark network of thin lines with pale white spots, canthal and loreal head stripes present with temporal and post-orbital stripes variably expressed and ventrum with little to moderate stippling.

*Description.* See Storr (1982) for a detailed description.

*Colour and pattern.* In life, background colour a uniform light grey to medium grey-brown with purplish hue. A relatively dense network of fine blackish-brown lines over upper surfaces including limbs and head; numerous fine pale white spots usually in contact with dark lines on the posterior edge. Dark brown canthal and loreal stripes usually visible, loreal stripe continuing posterior to eye as temporal stripe, upper and lower post-orbital stripes variably expressed. Original tails as for dorsum.

In preservative, background colour light grey to brown, with the purplish hues lost. The dark network and white spots highly subdued with many specimens having almost no pattern. Ventral surfaces a pale off-white to cream.

*Distribution.* Widely distributed throughout the Australian arid zone. In Western Australia, occurs in all the major interior deserts, but is absent from the Pilbara, Gascoyne, upper Murchison and Wheatbelt regions (Fig. 1C). It occurs throughout the Northern Territory, except for the northern Top End, and extends just over the Queensland border. In South Australia it occurs throughout the state except the southern corners.

*Habitat and ecology.* Arboreal, on shrubs, trees and logs throughout arid woodlands and dunes.

*Etymology.* The specific name *purpurascens* is Latin for ‘purplish’ and refers to the this species’ appearance in life.

*Comparisons with other species.* This species is one of the largest in the arid clade, reaching a maximum size of 67 mm SVL (all other species <58 mm). However, subadults and juveniles of this species may resemble others in the arid clade. It differs from *G. pilbara* and G. minuta by having a greyish-brown (vs reddish-orange) colouration. It further differs in snout length from *G. pilbara* (moderate length vs short) and *G. minuta* (vs long). From the geographically restricted *G. unguiculata* sp. nov. it differs by much larger body size in adults (average of 53.6 vs 37.3 mm SVL), lacking crescent-shaped markings on the dorsum and canthal and loreal stripes present (vs poorly defined).

*Gehyra purpurascens* most closely resembles *G. variegata*, *G. montium*, *G. versicolor*, *G. crypta* sp. nov. and *G. incognita* sp. nov., but differs in having a purplish tinge on the dorsum in life, lower pre-cloacal pore counts (8–11 vs 10–16) and for some comparisons often possessing three chin shields with the 3^rd^ infralabial notched.

*Remarks.* It is worth noting that *G. purpurascens* is both the largest member of the arid clade of the *variegata* group, and also the most widely distributed. Based on the small to moderate size of other members of the arid clade, the increase in size is likely to be a derived trait. However, *G. purpurascens* diverged in the late Miocene (Ashman et al., submitted) from its two closest relatives: the putative arboreal *G. incognita* sp. nov. in the northern Pilbara and *G. einasleighensis*, a rock-dwelling species from northern Queensland. Given the long divergence times, speculation into the evolution of the *purpurascens* species complex is rendered more difficult than more recent divergences observed in the *variegata* species complex.
