# Supplementary material for: Conspicuously concealed: revision of the arid clade of the Gehyra variegata (Gekkonidae) group in Western Australia using an integrative molecular and morphological approach, with the description of five cryptic species
Source: PeerJ. 2018 Jul 19;6:e5334. doi: 10.7717/peerj.5334 (PMC6054870; doi:10.7717/peerj.5334)
Supplement: Supplemental Information 3 — All specimens are from the Western Australian Museum (R prefixes omitted from the registration numbers). ‘Genotyped’ means mtDNA was sequenced, and ‘Morphology’ refers to specimens included in the morphological analyses. [file peerj-06-5334-s003.docx]

**Table S2. Morphological summary.** Covers all species treated herein, divided by sex (M or F). Sample sizes are provided after the species name and sex. Abbreviations and definitions of characters: SVL (snout-vent length) – from tip of snout to anterior edge of cloaca; TailL (tail length) – from posterior edge of cloaca to tip of tail, original tails only; HeadL (head length) – measured obliquely from tip of snout to retroarticular process at edge of jaw; HeadW (head width) – measured at widest part of head between eyes and ears; HeadD (head depth) – measured between eyes and ears; OrbitL (orbit length) – measured horizontally at widest point; EyeNarL (eye to naris length) – measured obliquely from anterior corner of eye to centre of nostril; SnoutEyeL (snout to eye length) – measured obliquely from anterior corner of eye to tip of snout; InterOrbL (interorbital length) – from anterior corners of eyes; InterNarL (internarial length) – measured from centre of each nostril; LegL (leg length) – from knee to heel; SupLab (supralabials), InfLab (infralabials) – enlarged scales counted until size reached that of normal background scales; Internarials – number of small scales between enlarged supranasals; 4TLam (fourth toe lamellae) – number of enlarged lamellae on the underside of the fourth toe; Pre-cloacal pores – number of pores anterior to cloaca in males, pore perforating centre of enlarged scales.

|  | *G. variegata*, M, 15 | *G. variegata*, F, 16 | *G. pilbara*, M, 14 | *G. pilbara*, F, 16 | *G. montium*, M, 15 | *G. montium*, F, 15 | G. purpurascens, M, 19 | G. purpurascens, F, 11 | *G. versicolor,* M, 2 | *G. versicolor,* F, 6 | *G. minuta*, M, 10 | *G. minuta*, F, 13 | *G. capensis* sp. nov., M, 15 | *G. capensis* sp. nov., F, 10 | *G. crypta* sp. nov., M, 13 | *G. crypta* sp. nov., F, 17 | *G. ocellata* sp. nov., M, 9 | *G. ocellata* sp. nov., F, 9 | *G. incognita* sp. nov., M, 9 | *G. incognita* sp. nov., F, 15 | *G. unguiculata* sp. nov., M, 4 | *G. unguiculata* sp. nov., F, 5 |
| --- | --- | --- | --- | --- | --- | --- | --- | --- | --- | --- | --- | --- | --- | --- | --- | --- | --- | --- | --- | --- | --- | --- |
| **SVL** | 43.5±1.1  (37.0–52.5) | 43.6±0.6  (39.5–49.0) | 45±1.1  (35.0–51.5) | 41.7±0.9  (33.5–46.5) | 46.0±0.7  (41.0– 49.5) | 42.8±1.0  (35.5– 48.0) | 55.2±1.3  (45.5–67.0) | 50.7±0.7  (48.0–57.0) | 51.0±0.5  (50.5–51.5) | 49.0±2.7  (40.5–58.0) | 36.9±0.8  (32.0–40.0) | 32.5±1.4  (24.0–39.0) | 41.4±0.7  (35.5–46.0) | 38.5±1.1  (34.0–44.5) | 47.7±0.6  (43.0–51.0) | 47.8±0.9  (41.0–56.5) | 40.6±1.7  (32.0–49.0) | 40.5±1.2  (37.0–49.0) | 44.9±1.7  (36.0–52.0) | 44.0±1.5  (25.0–49.0) | 38.5±0.3  (38.0–39.0) | 36.3±0.9  (34.5–39.0) |
| **TailL** | 47.3±2.4  (44.0–52.0) |  | 45.5±3.5  (42.0–49.0) | 43.0±2.7  (36.0–49.0) | 51.9±1.7  (47.0–57.0) | 44.8±2.1  (40.0–50.0) |  | 55.7±1.2  (54.0–58.0) | 56.0±0.0  (56.0–56.0) |  | 37.0±0  (37.0–37.0) | 29.0±8.0  (21.0–37.0) |  | 45.0±0  (45.0–45.0) | 57.0±1.0  (55.0–58.0) | 54.0±4.0  (50.0–58.0) | 36.0±0.0  (36.0–36.0) | 40.0±0.0  (40.0–40.0) |  | 42.0±0.0  (42.0–42.0) | 43.5±1.5  (42.0–45.0) |  |
| **HeadL** | 12.0±0.3  (9.4–14.0) | 11.6±0.2  (10.3–12.6) | 11.3±0.2  (9.4–12.3) | 10.5±0.2  (9.4–11.7) | 12.5±0.2  (11.4–13.6) | 11.4±0.3  (9.9–12.8) | 15.4±0.4  (13.1–18.8) | 14.0±0.3  (12.1–16.1) | 14.1±0.7  (13.4–14.9) | 13.3±0.6  (11.1–15.1) | 10.4±0.2  (9.8–11.3) | 9.1±0.3  (7.4–10.4) | 11.6±0.1  (10.6–12.2) | 11.2±0.3  (9.7–12.3) | 12.6±0.2  (11.3–14.2) | 12.3±0.2  (10.8–14.3) | 11.3±0.4  (9.6–12.9) | 11.1±0.3  (9.9–13.2) | 12.4±0.5  (9.8–14.6) | 12.1±0.4  (7.7–13.2) | 10.2±0.3  (9.4–10.8) | 9.8±0.2  (9.1–10.3) |
| **HeadW** | 8.0±0.2  (6.8–9.7) | 7.8±0.2  (6.4–9.3) | 7.9±0.2  (6.3–9.3) | 7.2±0.2  (5.9–8.5) | 8.2±0.2  (7.1–9.4) | 7.3±0.2  (6.0–8.4) | 10.2±0.3  (8.4–13.3) | 9.2±0.3  (7.8–10.6) | 9.9±0.3  (9.6–10.2) | 9.4±0.6  (7.0–11.6) | 6.9±0.1  (6.3–7.4) | 6.0±0.2  (4.4–7.2) | 7.7±0.1  (6.7–8.8) | 7.2±0.2  (5.9–8.1) | 8.5±0.2  (7.1–9.0) | 8.1±0.2  (6.6–9.7) | 7.7±0.4  (6.1–9.5) | 7.5±0.3  (6.7–9.9) | 8.5±0.4  (6.6–10.3) | 8.2±0.3  (4.8–9.3) | 7.0±0.2  (6.6–7.2) | 6.3±0.2  (5.8–6.9) |
| **HeadD** | 4.6±0.1  (3.5–5.4) | 4.4±0.1  (3.6–5.1) | 4.7±0.1  (4.0–5.4) | 4.2±0.1  (3.4–5.0) | 4.6±0.1  (3.9–5.3) | 3.9±0.1  (3.1–4.5) | 6.0±0.2  (4.9–7.5) | 5.1±0.2  (4.5–6.2) | 5.4±0.3  (5.1–5.6) | 5.0±0.2  (4.0–5.4) | 3.8±0.1  (3.3–4.5) | 3.3±0.1  (2.6–4.0) | 4.6±0.1  (3.6–5.2) | 4.1±0.1  (3.5–4.7) | 4.7±0.1  (4.2–5.1) | 4.4±0.1  (3.7–5.4) | 4.8±0.2  (3.7–5.6) | 4.5±0.2  (3.6–5.7) | 5.1±0.3  (3.8–6.3) | 4.6±0.2  (2.9–5.5) | 3.4±0.2  (2.9–3.8) | 3.1±0.2  (2.6–3.4) |
| **OrbitL** | 2.62±0.07  (2.18–3.27) | 2.66±0.07  (2.26–3.24) | 2.81±0.06  (2.43–3.26) | 2.70±0.05  (2.32–3.06) | 2.88±0.07  (2.44–3.34) | 2.64±0.08  (2.12–3.22) | 3.19±0.05  (2.58–3.45) | 3.10±0.09  (2.71–3.59) | 3.28±0.02  (3.26–3.29) | 2.96±0.11  (2.47–3.23) | 2.54±0.03  (2.40–2.67) | 2.34±0.08  (1.89–2.71) | 2.86±0.03  (2.67–3.05) | 2.73±0.09  (2.12–3.05) | 2.86±0.04  (2.52–3.07) | 2.86±0.08  (2.4–3.46) | 2.63±0.10  (2.24–3.1) | 2.62±0.09  (2.34–3.15) | 2.68±0.07  (2.42–3.07) | 2.64±0.07  (2.13–2.96) | 2.58±0.08  (2.43–2.77) | 2.44±0.10  (2.14–2.64) |
| **EyeNarL** | 3.56±0.10  (2.89–4.22) | 3.70±0.06  (3.15–4.26) | 3.31±0.08  (2.51–3.86) | 3.07±0.06  (2.52–3.40) | 3.83±0.07  (3.40–4.33) | 3.62±0.08  (3.08–4.17) | 4.87±0.12  (4.13–6.24) | 4.54±0.11  (3.99–5.15) | 4.28±0.22  (4.06–4.49) | 4.20±0.18  (3.50–4.79) | 3.14±0.05  (2.85–3.32) | 2.76±0.11  (2.08–3.22) | 3.66±0.04  (3.28–3.85) | 3.48±0.08  (3.02–3.89) | 4.05±0.10  (3.46–4.69) | 3.89±0.08  (3.28–4.54) | 3.37±0.12  (3.00–3.81) | 3.28±0.10  (2.98–3.81) | 3.89±0.15  (3.12–4.65) | 3.76±0.11  (2.44–4.41) | 3.01±0.16  (2.58–3.32) | 2.92±0.18  (2.49–3.39) |
| **SnoutEyeL** | 4.79±0.12  (4.10–5.59) | 4.82±0.10  (4.23–5.48) | 4.4±0.11  (3.71–4.98) | 4.25±0.07  (3.52–4.71) | 5.07±0.09  (4.27–5.51) | 4.74±0.12  (4.01–5.32) | 6.22±0.15  (5.05–7.67) | 5.75±0.14  (4.97–6.42) | 5.62±0.08  (5.54–5.69) | 5.62±0.23  (4.74–6.34) | 4.34±0.09  (3.91–4.83) | 3.89±0.13  (3.14–4.51) | 4.91±0.06  (4.38–5.29) | 4.75±0.14  (4.12–5.37) | 5.30±0.08  (4.85–5.72) | 5.16±0.11  (4.45–5.98) | 4.62±0.13  (3.93–5.15) | 4.53±0.15  (3.89–5.38) | 5.03±0.16  (4.38–5.74) | 4.92±0.14  (3.22–5.49) | 4.21±0.07  (4.14–4.42) | 4.23±0.13  (3.81–4.6) |
| **InterOrbL** | 4.15±0.09  (3.60–4.8) | 3.96±0.1  (3.20–4.55) | 3.4±0.09  (2.99–4.24) | 3.18±0.11  (2.21–3.66) | 4.06±0.06  (3.66–4.38) | 3.73±0.08  (3.07–4.16) | 5.00±0.13  (4.06–6.02) | 4.39±0.13  (3.92–5.39) | 4.32±0.04  (4.28–4.36) | 4.52±0.25  (3.47–5.24) | 3.47±0.10  (2.82–3.82) | 3.11±0.13  (2.14–3.68) | 4.18±0.11  (3.28–4.70) | 3.84±0.14  (3.08–4.38) | 4.09±0.09  (3.57–4.61) | 4.09±0.09  (3.55–4.84) | 3.70±0.13  (3.04–4.35) | 3.54±0.11  (3.15–4.25) | 4.34±0.20  (3.65–5.22) | 4.16±0.14  (2.98–4.80) | 3.41±0.09  (3.19–3.61) | 3.12±0.11  (2.85–3.37) |
| **InterNarL** | 1.63±0.05  (1.31–2.00) | 1.57±0.02  (1.40–1.68) | 1.46±0.05  (1.08–1.75) | 1.47±0.05  (1.00–1.72) | 1.61±0.03  (1.38–1.73) | 1.58±0.05  (1.21–1.86) | 2.04±0.06  (1.43–2.63) | 1.90±0.07  (1.58–2.43) | 1.79±0.09  (1.70–1.87) | 1.87±0.07  (1.54–2.07) | 1.36±0.03  (1.18–1.59) | 1.25±0.04  (1.03–1.44) | 1.50±0.04  (1.22–1.69) | 1.52±0.07  (1.22–1.84) | 1.72±0.03  (1.51–1.86) | 1.72±0.04  (1.50–2.13) | 1.43±0.07  (1.12–1.77) | 1.29±0.06  (1.09–1.67) | 1.78±0.07  (1.49–2.18) | 1.67±0.05  (1.06–1.94) | 1.32±0.01  (1.29–1.34) | 1.31±0.03  (1.23–1.38) |
| **LegL** | 5.9±0.2  (4.8–6.9) | 5.9±0.1  (5.5–6.4) | 6.5±0.2  (5.5–7.7) | 6.0±0.1  (4.6–6.9) | 6.1±0.1  (5.2–6.6) | 5.6±0.2  (4.2–6.3) | 7.5±0.2  (5.7–8.9) | 7.1±0.2  (6.4–8.6) | 7.1±0.2  (6.9–7.4) | 6.6±0.3  (5.4–8.0) | 5.2±0.1  (4.7–5.7) | 4.5±0.2  (3.3–5.3) | 5.8±0.1  (5.1–6.5) | 5.7±0.2  (5.0–6.4) | 6.4±0.1  (5.9–7.1) | 6.2±0.2  (5.4–7.5) | 6.1±0.3  (4.7–7.4) | 5.9±0.2  (5.2–7.3) | 6.6±0.3  (5.3–7.6) | 6.3±0.2  (3.9–6.9) | 5.1±0.1  (4.8–5.4) | 5.0±0.2  (4.5–5.6) |
| **SupLab** | 8.6±0.1  (8–9) | 8.3±0.1  (7–9) | 7.4±0.1  (7–8) | 7.8±0.1  (7–9) | 8.7±0.2  (8–10) | 8.5±0.2  (7–10) | 9.3±0.1  (9–10) | 9.5±0.2  (9–11) | 8.5±0.5  (8–9) | 9.5±0.3  (9–11) | 8.0±0.2  (7–9) | 7.9±0.2  (7–9) | 8.5±0.1  (8–9) | 8.2±0.1  (8–9) | 8.8±0.2  (8–10) | 8.5±0.2  (8–10) | 7.4±0.2  (7–8) | 7.3±0.2  (6–8) | 8.3±0.2  (8–9) | 8.7±0.1  (8–9) | 8.0±0.0  (8–8) | 7.4±0.2  (7–8) |
| **InfLab** | 7.8±0.2  (6–9) | 7.8±0.2  (6–9) | 6.9±0.1  (6–8) | 6.6±0.1  (6–7) | 8.3±0.1  (8–9) | 8.1±0.2  (7–9) | 8.4±0.1  (8–10) | 8.9±0.2  (8–10) | 8.0±1.0  (7–9) | 8.7±0.2  (8–9) | 7.4±0.2  (6–8) | 7.0±0.2  (6–8) | 7.5±0.17  (7–9) | 7.6±0.3  (7–9) | 8.0±0.1  (7–9) | 7.9±0.1  (7–9) | 6.8±0.2  (6–8) | 6.8±0.3  (6–8) | 7.3±0.2  (7–8) | 7.7±0.2  (7–9) | 7.5±0.3  (7–8) | 7.0±0.0  (7–7) |
| **Internarials** | 0.5±0.1  (0–1) | 0.5±0.1  (0–1) | 0.4±0.1  (0–1) | 0.4±0.1  (0–1) | 0.9±0.1  (0–1) | 0.9±0.1  (0–1) | 0.8±0.1  (0–1) | 0.8±0.1  (0–1) | 1.0±0.0  (1–1) | 0.5±0.2  (0–1) | 0.6±0.2  (0–1) | 0.8±0.1  (0–1) | 0.7±0.1  (0–1) | 0.8±0.1  (0–1) | 0.8±0.1  (0–1) | 0.8±0.1  (0–1) | 0.2±0.1  (0–1) | 0.6±0.2  (0–1) | 0.4±0.2  (0–1) | 0.6±0.1  (0–1) | 0.3±0.3  (0–1) | 1.0±0.0  (1–1) |
| **4TLam** | 6.5±0.2  (6–8) | 6.1±0.1  (6–7) | 7.0±0.1  (6–8) | 6.9±0.1  (6–7) | 6.8±0.1  (6–7) | 6.7±0.1  (6–7) | 7.4±0.1  (7–8) | 7.5±0.2  (6–8) | 7.0±0.0  (7–7) | 7.0±0.0  (7–7) | 6.3±0.2  (6–7) | 6.6±0.1  (6–7) | 6.3±0.1  (6–7) | 6.3±0.2  (6–7) | 6.6±0.2  (6–8) | 6.5±0.1  (6–7) | 6.2±0.1  (6–7) | 6.1±0.1  (6–7) | 6.1±0.1  (6–7) | 5.9±0.1  (5–6) | 6.0±0.0  (6–6) | 6.2±0.2  (6–7) |
| **Pre-cloacal pores** | 11.5±0.4  (10–15) |  | 12.5±0.3  (10–14) |  | 12.0±0.4  (10–15) |  | 9.7±0.2  (8–11) |  | 14.0±1.0  (13–15) |  | 12.0±0.3  (10–14) |  | 10.8±0.3  (9–12) |  | 12.4±0.4  (10–16) |  | 11.1±0.3  (10–12) |  | 12.0±0.6  (10–16) |  | 12.3±0.5  (11–13) |  |
| **HeadL/SVL** | 0.275±0.003  (0.253–0.291) | 0.266±0.004  (0.246–0.301) | 0.253±0.003  (0.237–0.270) | 0.254±0.004  (0.219–0.287) | 0.272±0.002  (0.260–0.283) | 0.267±0.003  (0.245–0.289) | 0.278±0.002  (0.262–0.292) | 0.276±0.005  (0.246–0.307) | 0.277±0.012  (0.265–0.288) | 0.273±0.004  (0.260–0.293) | 0.283±0.003  (0.265–0.305) | 0.281±0.004  (0.263–0.307) | 0.282±0.004  (0.256–0.307) | 0.291±0.006  (0.271–0.315) | 0.264±0.003  (0.252–0.287) | 0.258±0.002  (0.242–0.274) | 0.280±0.004  (0.262–0.301) | 0.274±0.002  (0.267–0.282) | 0.276±0.004  (0.254–0.293) | 0.277±0.003  (0.254–0.308) | 0.266±0.006  (0.248–0.277) | 0.271±0.005  (0.257–0.285) |
| **HeadW/SVL** | 0.185±0.002  (0.173–0.200) | 0.178±0.004  (0.158–0.206) | 0.177±0.003  (0.161–0.200) | 0.172±0.002  (0.156–0.186) | 0.178±0.003  (0.163–0.204) | 0.171±0.002  (0.154–0.188) | 0.184±0.002  (0.168–0.198) | 0.182±0.005  (0.159–0.215) | 0.194±0.004  (0.190–0.197) | 0.192±0.005  (0.173–0.206) | 0.187±0.004  (0.173–0.213) | 0.185±0.002  (0.172–0.203) | 0.187±0.002  (0.173–0.208) | 0.187±0.003  (0.164–0.201) | 0.178±0.003  (0.164–0.191) | 0.169±0.002  (0.153–0.182) | 0.189±0.002  (0.177–0.203) | 0.186±0.005  (0.168–0.211) | 0.190±0.004  (0.176–0.209) | 0.187±0.004  (0.169–0.216) | 0.181±0.004  (0.174–0.189) | 0.174±0.002  (0.167–0.178) |
| **HeadD/SVL** | 0.107±0.002  (0.093–0.121) | 0.101±0.002  (0.084–0.114) | 0.105±0.002  (0.095–0.118) | 0.102±0.003  (0.079–0.119) | 0.099±0.002  (0.083–0.111) | 0.092±0.002  (0.077–0.103) | 0.109±0.002  (0.091–0.121) | 0.101±0.003  (0.090–0.115) | 0.106±0.004  (0.102–0.110) | 0.102±0.003  (0.094–0.117) | 0.104±0.003  (0.091–0.117) | 0.102±0.002  (0.091–0.112) | 0.110±0.001  (0.101–0.118) | 0.106±0.003  (0.087–0.119) | 0.098±0.002  (0.085–0.109) | 0.092±0.002  (0.081–0.101) | 0.117±0.003  (0.104–0.131) | 0.112±0.003  (0.098–0.124) | 0.113±0.004  (0.091–0.129) | 0.104±0.003  (0.080–0.120) | 0.087±0.004  (0.076–0.097) | 0.084±0.003  (0.074–0.089) |
| **SnoutEyeL/SVL** | 0.110±0.001  (0.102–0.117) | 0.111±0.002  (0.100–0.127) | 0.098±0.002  (0.085–0.107) | 0.102±0.002  (0.093–0.119) | 0.110±0.001  (0.100–0.121) | 0.111±0.002  (0.098–0.120) | 0.113±0.002  (0.104–0.132) | 0.113±0.003  (0.101–0.133) | 0.110±0.000  (0.110–0.110) | 0.115±0.003  (0.109–0.123) | 0.118±0.002  (0.109–0.128) | 0.120±0.002  (0.109–0.134) | 0.119±0.002  (0.102–0.129) | 0.124±0.002  (0.114–0.131) | 0.111±0.001  (0.103–0.120) | 0.108±0.001  (0.102–0.118) | 0.115±0.003  (0.100–0.123) | 0.112±0.001  (0.105–0.119) | 0.112±0.002  (0.099–0.122) | 0.112±0.002  (0.102–0.129) | 0.109±0.002  (0.106–0.116) | 0.117±0.002  (0.110–0.120) |
| **SnoutEyeL/HeadL** | 0.401±0.005  (0.378 –0.439) | 0.417±0.005  (0.386 –0.448) | 0.388±0.006  (0.339 –0.435) | 0.404±0.005  (0.370 –0.434) | 0.406±0.006  (0.373 –0.464) | 0.415±0.006  (0.380 –0.483) | 0.405±0.005  (0.368 –0.456) | 0.412±0.004  (0.388 –0.431) | 0.399±0.015  (0.383 –0.414) | 0.423±0.009  (0.400 –0.463) | 0.416±0.007  (0.377 –0.456) | 0.429±0.006  (0.394 –0.462) | 0.422±0.005  (0.388–0.463) | 0.425±0.006  (0.397 –0.460) | 0.421±0.005  (0.396 –0.446) | 0.419±0.004  (0.392 –0.463) | 0.409±0.007  (0.367 –0.432) | 0.408±0.005  (0.391 –0.432) | 0.407±0.007  (0.381 –0.447) | 0.406±0.006  (0.362 –0.444) | 0.412±0.013  (0.383 –0.439) | 0.430±0.007  (0.420 –0.459) |
| **TailL/SVL** | 1.167±0.037  (1.114–1.238) |  | 1.151±0.049  (1.101–1.200) | 1.101±0.023  (1.043–1.140) | 1.114±0.016  (1.065–1.152) | 1.145±0.016  (1.111–1.179) |  | 1.045±0.018  (1.018–1.078) | 1.109±0.000  (1.109–1.109) |  | 1.028±0.000  (1.028–1.028) | 0.944±0.069  (0.875–1.014) |  | 1.154±0.000  (1.154–1.154) | 1.181±0.040  (1.137–1.261) | 1.150±0.013  (1.137–1.163) | 1.125±0.000  (1.125–1.125) | 1.067±0.000  (1.067–1.067) |  | 1.077±0.000  (1.077–1.077) | 1.131±0.054  (1.077–1.184) |  |
